# Revisiting the Neuroblastoma Cell-Based Assay (CBA-N2a) for the Improved Detection of Marine Toxins Active on Voltage Gated Sodium Channels (VGSCs)

**DOI:** 10.3390/toxins12050281

**Published:** 2020-04-27

**Authors:** Jérôme Viallon, Mireille Chinain, Hélène Taiana Darius

**Affiliations:** Institut Louis Malardé (ILM), Laboratory of Marine Biotoxins-UMR 241-EIO, 98713 Papeete-Tahiti, French Polynesia; jviallon@ilm.pf (J.V.); mchinain@ilm.pf (M.C.)

**Keywords:** CBA-N2a, standardization, matrix effects, absorbance data, ciguatoxins, brevetoxins, saxitoxins, biological sample, seafood safety

## Abstract

The neuroblastoma cell-based assay (CBA-N2a) is widely used for the detection of marine biotoxins in seafood products, yet a consensus protocol is still lacking. In this study, six key parameters of CBA-N2a were revisited: cell seeding densities, cell layer viability after 26 h growth, MTT incubation time, Ouabain and Veratridine treatment and solvent and matrix effects. A step-by-step protocol was defined identifying five viability controls for the validation of CBA-N2a results. Specific detection of two voltage gated sodium channel activators, pacific ciguatoxin (P-CTX3C) and brevetoxin (PbTx3) and two inhibitors, saxitoxin (STX) and decarbamoylsaxitoxin (dc-STX) was achieved, with EC_50_ values of 1.7 ± 0.35 pg/mL, 5.8 ± 0.9 ng/mL, 3 ± 0.5 ng/mL and 15.8 ± 3 ng/mL, respectively. When applied to the detection of ciguatoxin (CTX)-like toxicity in fish samples, limit of detection (LOD) and limit of quantification (LOQ) values were 0.031 ± 0.008 and 0.064 ± 0.016 ng P-CTX3C eq/g of flesh, respectively. Intra and inter-assays comparisons of viability controls, LOD, LOQ and toxicity in fish samples gave coefficients of variation (CVs) ranging from 3% to 29%. This improved test adaptable to either high throughput screening or composite toxicity estimation is a useful starting point for a standardization of the CBA-N2a in the field of marine toxin detection.

## 1. Introduction

The bio-accumulation of marine biotoxins produced by phytoplankton in filter-feeding invertebrates and finfish not only poses significant health threats to consumers but also has detrimental effects on the economies of nations highly dependent on seafood consumption for their subsistence [1,2,3]. Among these potent marine biotoxins are neurotoxins acting on the voltage gated sodium channels (VGSCs) of excitable cells, namely VGSC activators such as brevetoxins (PbTxs) and ciguatoxins (CTXs), and VGSC inhibitors such as saxitoxins (STXs) and tetrodotoxins (TTXs) [1,2]. Detection and quantification of these groups of marine toxins remain highly challenging due to the wide range of congeners present in trace amounts in contaminated biological matrices [4]. In addition, an official reference method is still lacking for most of these toxins due to the poor availability of certified reference standards [5]. Although the mouse bioassay has been recommended by the European Food Safety Authority (EFSA) for years until 2015, the European Union now strongly advocates the use of analytical techniques based on liquid chromatography coupled with mass spectrometry in tandem (LC-MS/MS) and high performance liquid chromatography with fluorescence detection (HPLC-FLD) for the detection of lipophilic and hydrophilic toxins, respectively [6,7,8,9,10]. However, the need for alternative high throughput methods for toxin screening and quantification purposes remains [8,11]. Among the readily available and most widely used in vitro methods, the functional assay known as the cell-based assay (CBA) that uses a neuroblastoma (N2a) cell line appears as the most promising one [2,12].

Based on the mode of action of VGSC toxins, the CBA-N2a was initially developed to detect two VGSC inhibitors, STXs and TTXs [13]. Those marine toxins have no cytotoxic effect on N2a cells and their detection requires the addition of the sodium/potassium (Na^+^/K^+^) ATPase pump blocker ouabain (O), together with the sodium-channel activator veratridine (V), which induces permanent activation of the VGSCs [14]. Under this O/V treatment (OV^+^ conditions), sodium influx resulted in the cellular swelling and subsequent death of N2a cells [13]. Following the addition of STX or TTX, an increase of cell viability is observed as the two inhibitors counteract the effect of ouabain and veratridine. At that time, viability measure was achieved by a visual estimation of the morphological changes of N2a cells and the enumeration of viable cells under an inverted microscope. This assay performed in a 96-well microplate format has been applied to the detection of Paralytic Shellfish Poisoning (PSP) toxin standards [13] and to assess the presence of PSP toxins in biological extracts [15,16]. Standardization was also achieved using a certified STX standard for the detection of PSP toxins in shellfish and dinoflagellate cell extracts using automated endpoint determination with final readout based on crystal violet staining [17]. Further, this CBA-N2a was applied to detect VGSC inhibitors in marine bacterial supernatants using neutral red for final estimation of toxicity [18] and was subsequently extended to the detection of PbTxs and CTXs [19,20]. Contrary to VGSC inhibitors, these two VGSC activators induce a decrease in N2a cell viability in OV^+^ conditions [19,20], whereas no cytotoxic effect is observed in the absence of O/V treatment [21]. These same authors also introduced a more workable measure of cell viability using the 3-(4,5-dimethyl-2-thiazol)-2,5-diphenyl-2H-tetrazolium bromide (methylthiazolyldiphenyl-tetrazolium bromide) colorimetric assay (known as MTT assay), as modified from previous protocols aiming at assessing either cellular growth and survival [22] or chemosensitivity in established cell lines [23]. The MTT assay is widely used to measure cell viability and proliferation [24,25,26,27], and is based on the capacity of mitochondrial dehydrogenase enzymes present in living cells to reduce tetrazolium salts into an insoluble purple formazan product [28] localized in lipids droplets [29]. The readout step was first established using dimethyl-sulfoxide (DMSO) to dissolve formazan products [23], the amount of the formazan produced is directly linked to the number of viable cells remaining at the end of the assay. This estimation is achieved by means of an automated readout. Two patents describing an improved and simplified CBA-N2a were eventually filed, leading to great expectations among the scientific community that this bioassay could soon be routinely used to detect both activators and inhibitors of sodium channels [30,31]. However, neither commercial kit nor detailed protocol is available to date.

Briefly, the CBA-N2a originally set by Manger et al. [19,20] consists of three major steps; (i) cell seeding of 100,000 cells/200 µL/well in a 96-well microplate left to grown for ≈ 24 h, (ii) O/V treatment followed by cell layer exposure to toxin standards and/or biological samples during 24 to 48 h, depending on the targeted toxin activity (final reaction volume of 230 µL/well) and (iii) N2a cell viability readout at 570 nm using the MTT colorimetric assay. Practically, the detection of VGSC activators and inhibitors by CBA-N2a requires 3 and 4 days, respectively.

Since 1993, the CBA-N2a has been widely used in a number of studies for the detection of VGSC toxins and related toxin families. However, a comprehensive review of the literature shows that the protocol initially defined by Manger et al. [19,20] has undergone numerous changes: for instance, (i) up to 13 different cell seeding densities have been tested, ranging from 10,000 to 250,000 cells/well [32,33,34,35,36,37,38,39,40,41,42,43,44,45,46,47,48,49,50,51,52,53,54,55,56,57,58,59,60,61,62,63,64,65,66,67,68,69,70,71,72,73,74,75,76,77,78,79,80,81,82,83,84,85,86,87,88] suggesting that cell confluence likely varied substantially between studies, especially when estimated by eye measurement; (ii) the culture medium established for cell layer implementation, which formerly used 10% of fetal bovine serum (FBS), was often reduced to 5% in many studies [32,33,36,37,38,39,42,46,48,54,59,69,70,74,77,78,81,82,83,85,86,89]; (iii) the confluence level reached by N2a cells after 24 h growth varied from non-confluent cells [66] to >90% confluence [48,59,63,64,65,72,73,77,78,83] or even was not specified in some studies [33,34,35,36,37,38,40,41,42,43,47,49,50,51,52,53,54,55,56,57,58,61,66,67,69,70,74,75,76,79,80,81,82,85,87,88,89,90].

Other steps of the CBA-N2a have also been the subject of substantial modifications. For instance, although many studies used a 500/50 µM (1:10 ratio) for O/V treatment [32,33,34,35,36,37,38,39,40,41,42,43,44,45,46,47,48,49,76,89,91,92] as initially defined by Manger et al. [19,20,30,31], numerous changes in O/V concentrations [54,55,56,57,59,61,63,64,65,71,72,73,74,75,77,78,79,80,81,82,83,85,87,93,94,95], O/V ratios [50,51,52,69,86] and reaction volumes [32,33,35,36,49,51,53,59,61,63,64,69,73,75,77,78,79,80,84,86,87,88,89,90] were further proposed. Interestingly, in many of these studies, the effects on cell viability resulting from all these modifications were rarely addressed but, when reported, showed incongruent results. For instance, O/V treatment at 500/50 µM was shown to induce highly variable effects between 20–92% [34,45,68,96]. Conversely, similar effects (10–56%) were reportedly obtained at lower O/V concentrations [50,86]. The most significant modification was the use of two different O/V treatments, i.e., 100/10 and 300/30 µM, in order to induce 20% and 80% of cell mortality, respectively, for a more reliable assessment of the effects of VGSC toxin activators and inhibitors on N2a cells [83]. Several changes were also made to the final reaction volume (230 µL) at the last step of the CBA-N2a, i.e., cell exposure to toxin standards or biological samples, with volumes varying from 100 to 210 µL [32,33,34,35,36,45,49,51,53,59,61,63,64,69,73,75,77,78,79,80,84,86,87,88,89,90,95]. Moreover, measuring cell viability by means of the MTT colorimetric assay is classically performed at 570 nm, however, different wavelengths were also tested that ranged from 490 to 595 nm [33,35,36,38,39,40,41,42,45,50,56,57,61,64,67,68,69,71,75,76,79,80,84,85,87,88,90,93,97]. Finally, water-soluble tetrazolium reagents were sometimes preferred to the former water insoluble formazan product, with the use of WST-8 [49,60] or commercial kits such as CellTiter 96^®^ Aqueous One solution [33,38,69,86], Cell Counting Kit-8 [47] or XTT Cell Proliferation Assay kit [98].

This wide range of “in-house” methods described in the literature highlights the present lack of a consensus, standardized protocol for CBA-N2a, making any attempt to compare CBA data between assays and/or laboratories difficult or impossible. It also greatly hampers all current efforts to establish the CBA-N2a tool as a potential alternative reference method to LC-MS/MS. In this context, the present work aims at a comprehensive revisit of the CBA-N2a by evaluating the effects of six key parameters that are critical in obtaining reliable toxicity results, i.e., cell seeding densities, cell layer viability after 26 h growth, MTT incubation time, O/V treatment and solvent and matrix effects. To evaluate its robustness, the newly improved protocol thus established was further applied to the detection of toxins active on VGSC families (activators vs. inhibitors), and the estimation of CTX-like toxicity in fish samples of known ciguatoxic status.

## 2. Results

### 2.1. Characterization of N2a Cell Growth and Initial Viability

**Figure 1:**
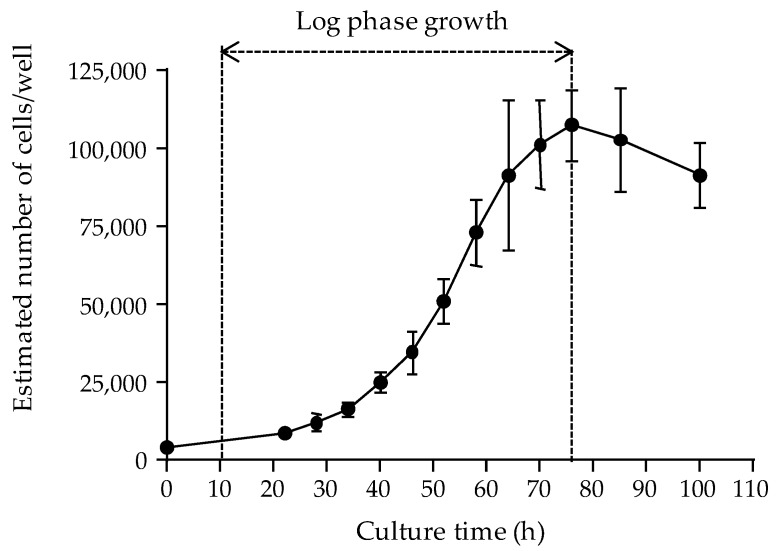
Characteristic growth pattern of the neuroblastoma (N2a) cell line used in this study. Densities in 96-well microplates were extrapolated from growth experiments conducted over a 4 days period in 25 cm^2^ culture flasks using a 10% fetal bovine serum (FBS) culture medium. Data represent the mean ± standard deviation (SD) of one experiment (N2a cells at 383 passages (P)), with *n* = 10 counts for each point. Coefficients of variation (CV) ranged from 10.8% to 26.3%.

The N2a CLL 131 cell line used in this study displayed a typical growth curve characterized by a short lag phase (slow cell growth), followed by a log phase (exponential proliferation of cells and consumption of nutrients of the culture medium) until a maximum density of ≈ 100,000 cells/well is reached, and a late stationary/senescence phase (reduced cell proliferation) (Figure 1).

The linearization of the log phase of the growth curve was defined by the following equation:

Y = 0.0578X + 7.7948 (r^2^ = 0.9974) (1)

in which X is the culture time (hours) and Y is the Ln-transformed cell number. Based on this equation, it was concluded that the N2a growth curve was characterized by a 9.8 h lag phase (Y = Ln (4288)) and that the cell number increased by two-fold after an additional 12 h. Moreover, a cell seeding density of 50,000 ± 10,000 cells/well allowed reaching a maximum cell density of ≈ 100,000 cells/well after 22 h culture time. For more convenience, a culture time of 26 h post-seeding was selected in all further experiments.

**Figure 2:**
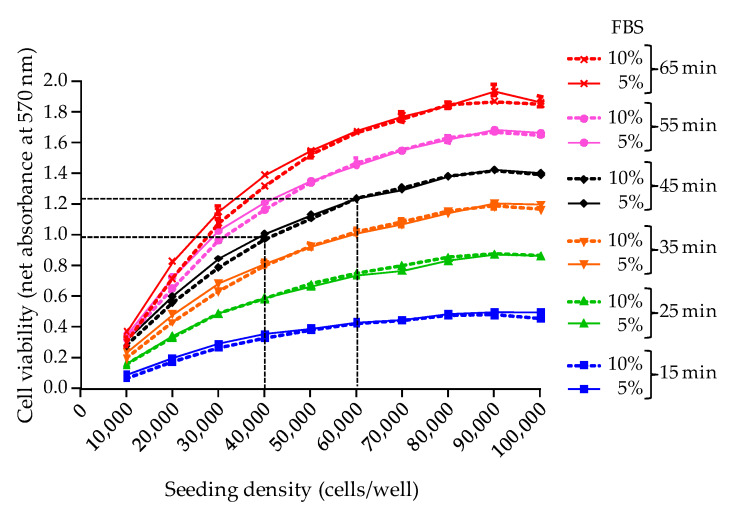
Initial viability of N2a cells observed in 96-well microplates after 26 h growth in 5% FBS (full line) and 10% FBS (dotted line) culture medium, at different cell seeding density. Six distinct MTT incubation times were also tested: 15 min (blue); 25 min (green); 35 min (orange); 45 min (black); 55 min (pink); 65 min (red). Data represent the mean ± SD of one microplate (N2a cells at 536 P), each point tested in six wells. Mean CVs were <3%. Absorbance values were measured at 570 nm via the MTT assay.

Next, the N2a cell initial viability after 26 h growth was compared at ten different cell seeding densities ranging from 10,000 to 100,000 cells/well and in two culture conditions (5% and 10% FBS growth medium) (Figure 2). Results showed that (i) the highest absorbance value is consistently obtained at a cell seeding density around the maximum number of cells supported by microplate wells, and (ii) absorbance values increase in proportion with MTT incubation times (Figure 2). For instance, the maximum absorbance value measured after 26 h culture time and 45 min MTT incubation time was 1.4 while selecting a cell seeding density of 50,000 ± 10,000 cells/well (vertical dotted lines, Figure 2) allowed to reach absorbance values comprised between 1 and 1.25 (horizontal dotted lines, Figure 2). This absorbance range was considered optimal when the detection of a decrease in cell viability is sought. Finally, no differences were observed when using either 5% or 10% FBS growth medium, which indicates possible saving opportunities on this expensive reagent at this step of the CBA-N2a.

Based on these results, all further CBA-N2a experiments were conducted as follows:

- Implement cell layer in microplates using a cell seeding density of 50,000 ± 10,000 cells/well in 200 µL of a 5% FBS culture medium, in order to reach a maximum cell density of 100,000 ± 20,000 cells/well after ≥22 h of culture.
- Conduct the MTT assay at an incubation time of 45 min, in order to reach an absorbance value ≥1.0 that is used to define N2a “initial viability”.
- For each experiment, dedicate a separate microplate to measure the N2a cell initial viability after 26 h of growth defined as the “Reference Cell Viability” control (RCV control).

### 2.2. Characterization of N2a Cell Final Viability

The second step of CBA-N2a is the exposure of N2a cells to VGSC activators or inhibitors, in OV^−^ or OV^+^ conditions. Following an additional culture time of 19 h overnight, the final viability of N2a cells was assessed as previously described. 

#### 2.2.1. N2a Cell Final Viability in OV^−^ Conditions

**Figure 3:**
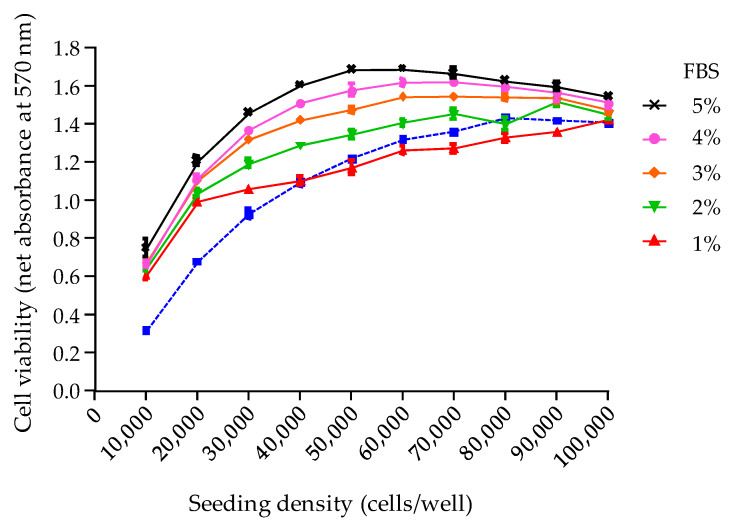
Final viability of N2a cells observed at different seeding densities when cultured 26 h in 5% FBS culture medium followed by 19 h in fresh growth medium supplemented with 1% FBS (red), 2% FBS (green), 3% FBS (orange), 4% FBS (pink) and 5% FBS (black). The initial viability measured in the Reference Cell Viability (RCV) control microplate after 26 h of growth is represented by the blue dotted curve. Data represent the mean ± SD of one microplate (N2a cells at 537 P), each point tested in six wells. Absorbance values were measured at 570 nm via the MTT assay, after a 45 min MTT incubation time.

A final cell viability lower than the initial cell viability (as measured in the RCV control) was observed only with 1% FBS growth medium at cell seeding density > 40,000 cells/well (Figure 3). Conversely, all other growth media allowed to reach a final viability higher than the one displayed by the RCV control, regardless of the cell seeding density (Figure 3), suggesting a complete renewal of the culture medium with 2% FBS growth medium is sufficient to ensure a stable cell viability during 19 h additional culture of the toxin exposure. A better stability of cell viability was consistently achieved at cell seeding densities > 40,000 cells/well. In other words, working at a cell seeding density of 50,000 ± 10,000 cells/well will allow to reach an absorbance value ≥1, close to the one measured in the RCV control (Figure 3).

Based on these results, the following procedure was selected to ensure that, at this step of the CBA-N2a, a final cell viability close to the initial cell viability is obtained in OV^−^ conditions:

- After cell layer implementation (26 h after seeding), remove the used culture medium from microplates (except for the RCV control plate which is sacrificed to get the initial N2a cell viability via the MTT assay).
- Replace with 200 µL of fresh 2% FBS growth medium prior to an additional culture time of 19 h.
- Measure the absorbance data in control wells (*n* = 3) in the absence of O/V treatment (COV^−^) to characterize the N2a cell final viability in OV^−^ conditions.
- Compare absorbance data in COV^−^ control vs. RCV control to verify cell viability is maintained in OV^−^ conditions.

#### 2.2.2. N2a cell Final Viability in OV^+^ Conditions

**Figure 4:**
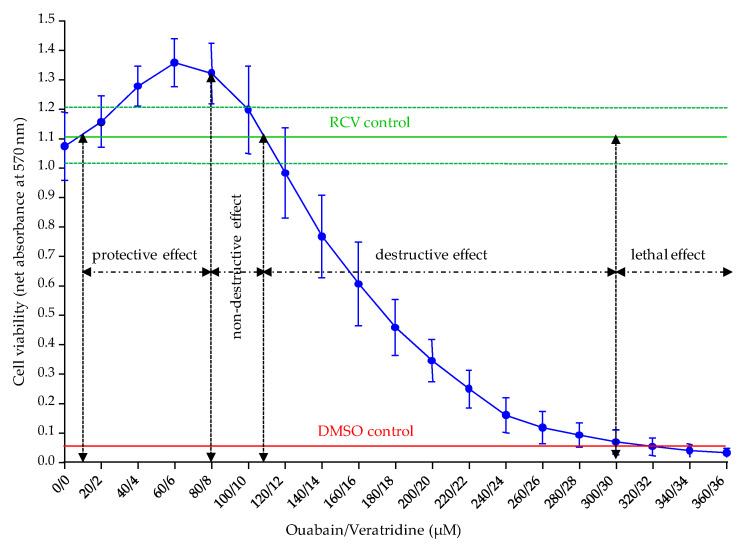
Dose-response curve of N2a cells when exposed to increasing concentrations of Ouabain and Veratridine (O/V) treatments ranging from 0/0 to 360/36 µM. Data represent the mean ± SD of one microplate in six independent experiments corresponding to cell passage numbers of 384, 542, 800, 801, 803 and 804 P, each point run in triplicate. The mean absorbance ± SD values corresponding to the RCV control (horizontal green line and dotted green lines) and DMSO control (horizontal red line) were determined at 1.105 ± 0.096 and 0.042 ± 0.001, respectively. Absorbance values were measured at 570 nm via the MTT assay, after a 45 min MTT incubation time. The four different effects induced by increasing concentrations of O/V treatment on N2a cells, i.e., protective, non-destructive, destructive and lethal are also represented.

As expected, absorbance data measured at 0/0 µM (OV^−^ conditions) was close to the one of the RCV control (Figure 4). At O/V concentrations ranging from 10/1 to 80/8 µM, a “protective effect” was observed as evidenced by an increase in cell viability of ≈ 20% above the RCV control (Figure 4). Between 80/8 and ≈ 110/11 µM, although a slight decrease in absorbance data was observed, these values were consistently found above the RCV control (Figure 4), suggesting a slightly toxic effect, although regarded as “non-destructive effect” of O/V treatment on N2a cells (Figure 4). At higher concentrations, however, cell viability decreased in a dose-dependent manner down to the level observed for DMSO control, which was indicative of a “destructive effect” of O/V treatment on N2a cells (Figure 4). Above 300/30 µM, the effect of O/V on cell viability was considered “lethal” with the complete elimination of cell viability (Figure 4). Concerning the variability of O/V treatment obtained across a wide range of N2a cell passages from 384 to 804 P, the CVs were below 12.4% for the “non-destructive” O/V treatment between 80/8 and 100/10 µM selected for the detection of VGSC activators. When seeking to detect VGSC inhibitors, the absorbance values were close to 0 showing non significative CVs above 45% for the “destructive” O/V treatment between 270/27 and 300/30 µM.

Based on these results, the O/V treatment conditions best adapted to the type of activity we seek to detect on target cells (e.g., activation or inhibition of VGSCs on N2a cells) were defined as follows:

- Select non-destructive O/V treatment conditions (between 80/8 and 100/10 µM) in order to induce a final cell viability slightly above the one observed in the RCV control, so that any loss in cell viability can be assigned to the specific activity of VGSCs activators. In the following experiments (Section 2.4.2 and Section 2.4.3), 100/10 and 85.7/8.57 µM (final concentrations) were used.
- Select destructive O/V treatment conditions close to the lethal effect (between 270/27 and 300/30 µM) in order to induce a final cell viability slightly above the one observed in the DMSO control, so that any increase in cell viability can be assigned to the specific activity of VGSC inhibitors. In the following experiments (Section 2.4.2), 270/27 µM (final concentrations) were used.
- Measure the absorbance data in control wells (*n* = 3) in the presence of O/V treatment (COV^+^) to characterize the N2a cell final viability in OV^+^ conditions.
- Compare absorbance data in COV^+^ control vs. RCV control to verify the efficiency of the O/V treatment conditions applied in the experiment.

### 2.3. Characterization of the Unspecific Effects of Solvent and Dry Extract on N2a Cell Viability

The issue of potential solvent toxicity and sample extract matrix effects on N2a cell viability was also taken into consideration.

#### 2.3.1. Solvent Effects

**Figure 5:**
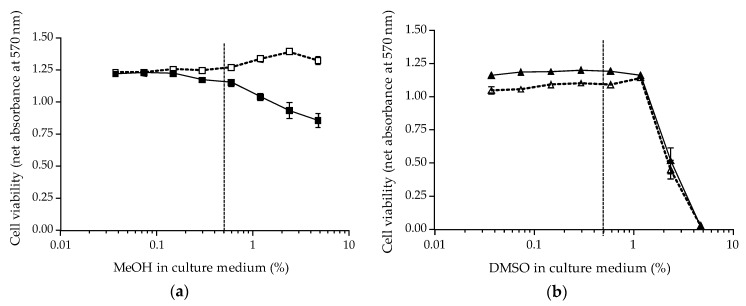
Dose-response curves of N2a cells when exposed to increasing concentrations of two solvents, in OV^−^ (open symbols) and OV^+^ (solid symbols) conditions at 100/10 µM (final concentrations); (**a**) MeOH (□/■) and (**b**) DMSO (∆/▲) were tested using N2a cells at 551 and 549 P, respectively. Data represent the mean ± SD of one microplate, with each point run in triplicate. Absorbance values were measured at 570 nm via the MTT assay, after a 45 min MTT incubation time. The initial cell viability in the RCV control was 1.139 ± 0.021 and 0.995 ± 0.031 and the final cell viability in the absence of O/V treatment (COV^−^) control was 1.217 ± 0.025 and 1.047 ± 0.023 for (**a**,**b**), respectively. The dotted vertical line corresponds to the maximum solvent concentration 0.5% for solvent interferences.

Using methanol (MeOH), a progressive loss in N2a cell viability is observed in 100/10 µM OV^+^ conditions at solvent concentrations ≥0.8%, giving a mean absorbance value ≈ 25% lower than the one measured in the RCV control at the highest concentration tested (Figure 5a). In OV^−^ conditions, an opposite effect is observed as evidenced by an increase in cell viability also at concentrations ≥0.8% (Figure 5a). Conversely, regardless of the OV conditions, DMSO consistently induced an abrupt decrease in cell viability at concentrations ≥1% until the complete elimination of viable N2a cells (Figure 5b). These findings indicate that both solvents can be used provided a concentration <0.8% is selected and that the maximum concentration of solvent can start at 0.5%. Practically, the use of non-volatile DMSO was preferred for the preparation of stock solutions (especially for toxin standards) as it is non-hazardous and likely to ensure stable toxin concentration over long periods of storage.

Consequently, a dilution of at least 1:200 giving the first final concentration (C1) of the toxin standard/sample extract stock solutions in MeOH or DMSO was achieved in all further CBA-N2a experiments, as follows:

- Initial dilution of at least 1:10 in 2% FBS culture medium,
- Final dilution at 1:21 by direct addition of 10 µL of initial dilution in 200 µL of growth medium. This will ensure the final solvent concentration in wells does not exceed 0.5%.

#### 2.3.2. Biological Matrix effects

**Figure 6:**
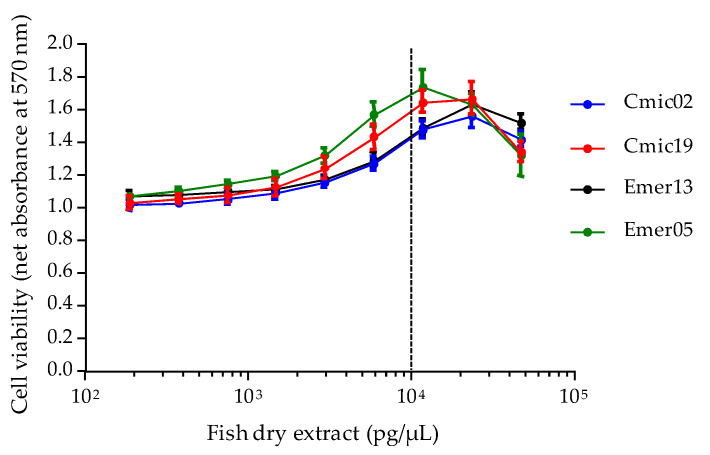
Dose-response curves of N2a cells in OV^−^ conditions when exposed to increasing concentrations of LF90/10 dry extracts prepared from *Chlorurus microrhinos* Cmic02 (blue), Cmic19 (red), *Epinephelus merra* Emer13 (black) and Emer05 (green) fish samples. Data represent the mean ± SD of three independent microplates (N2a cells at 800, 801 and 803 P), each point run in triplicate. Absorbance values were measured at 570 nm via the MTT assay, after a 45 min MTT incubation time. The cell viability in the RCV control was determined at 1.143 ± 0.009, while the final cell viability in COV^−^ control was 1.037 ± 0.053. The dotted vertical line corresponds to the maximum concentration of dry extract (MCE = 10,000 pg/µL) for matrix interferences.

In this study, the dry extract weights (DEW) obtained from 10 g of fish flesh ranged from 2.7 to 4.3 mg (Section 5.2), but they have been shown to sometimes vary up to ten-fold in other studies (data not shown). When tested at similar concentrations in OV^−^ conditions, all extracts induced a final cell viability slightly above the one observed in the RCV control at concentrations ≥1500 pg/µL, whereas cell viability could increase up to 70% as observed for sample Emer05 at 10,000 pg/µL (or 30.08 ± 5.92 µg fish flesh equivalent/µL) (Figure 6). Above this concentration, a decrease in cell viability rapidly occurred, suggesting an unspecific cytotoxicity on N2a cells likely due to matrix effects (Figure 6).

Based on these results, the MCE for LF90/10 fractions was set at 10,000 pg/µL for this study in all further experiments. Prior to CBA-N2a toxicity analysis, dry extract solutions of fish samples were thus prepared as follows:

- For all fish samples, resuspend LF90/10 dry extract in MeOH or DMSO at 10 mg/mL, then proceed with an intermediate 1:50 dilution of this previous stock solution.
- Add 10 µL of the solution thus obtained in 200 µL of growth medium to test the highest final concentration below the MCE, i.e., 9524 pg/µL, in the absence vs. under non-destructive O/V treatment.

This revisited CBA-N2a resulted in a practical guide that is presented in the Supplementary Materials.

### 2.4. Application to the Detection of VGSC Activators and Inhibitors

#### 2.4.1. N2a Cell Initial Viability

To test the relevance of the six parameters revisited in Section 2.1, Section 2.2 and Section 2.3, this improved protocol was further applied to the detection of different toxin standards acting on VGSCs. 

First, the initial viability of N2a cells prior to toxin exposure was assessed by measuring absorbance values in RCV control using the MTT colorimetric assay in three independent experiments. The metabolization of MTT into formazan by N2a viable cells resulted in a uniform blue color visible in all wells, proof that a high cell layer confluence was attained. Following the addition of DMSO and cell lysis, a dark purple color was released in each well (Figure S1).

The net absorbance data obtained for RCV controls in all three experiments were indicative of a high viability of N2a cells (mean value between 1 and 1.25) and were also highly reproducible, with CVs below 5% (Table 1).

#### 2.4.2. Detection of VGSC Activators and Inhibitors

In OV^−^ conditions (upper half of the microplates), the same dark purple color was observed for COV^−^ control and all concentrations tested regardless of the toxin standard (Figure S1). Conversely, in OV^+^ conditions (bottom half of the microplate), a progressive fading of this dark purple color was observed with increasing concentrations of P-CTX3C and PbTx3, until complete discoloration, signing the total death of N2a cells (Figure S1). Otherwise, a progressive darkening of the pale color observed at low concentrations occurred with increasing concentrations of STX and dc-STX, signing a progressive restoration of N2a cell viability (Figure S1). 

For VGSC activators, net absorbance values showed that COV^−^ and COV^+^ control absorbance data were close to that of the RVC control. The same observation applies to VGSC inhibitors except in OV^+^ conditions where absorbance data of COV^+^ controls were in the range of 0.1.

Inter-assay comparison in three independent experiments showed that net absorbance data are reproducible with CVs of 6.1% and 5.4% for COV^−^ controls, and 1.6% and 4.5% for COV^+^ controls of P-CTX3C and PbTx3, respectively. Likewise, net absorbance data of COV^−^ controls showed CVs of 6% and 3.9% of STX and dc-STX, respectively, but 41.4% and 37.5% for COV^+^ controls, these latter values being close to 0 absorbance. 

**Figure 7:**
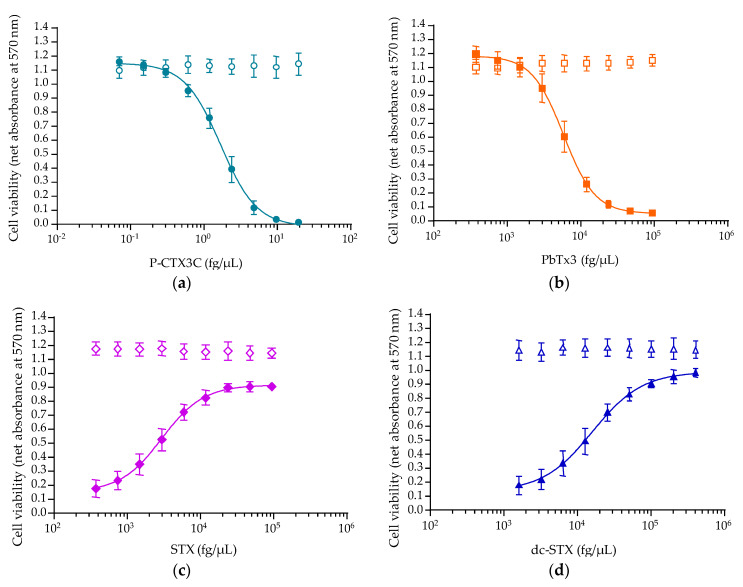
Dose-response curves displayed by N2a cells when exposed to nine increasing concentrations of toxin standards after 19 h exposure time, in OV^−^ (open symbols) and OV^+^ (solid symbols) conditions at 100/10 µM for voltage gated sodium channels (VGSC) activators and 270/27 µM for VGSC inhibitors (plain symbols): (**a**) P-CTX3C (○/●); (**b**) PbTx3 (□/■); (**c**) (STX) (◊/♦); (**d**) dc-STX (∆/▲). Data represent the mean ± SD of one microplate in three independent experiments (N2a cells at 662, 663 and 666 P), with each concentration run in triplicate. Net absorbance values were 1.101 ± 0.067 and 1.077 ± 0.058 in COV^−^ controls (*n* = 3), and 1.209 ± 0.014 and 1.226 ± 0.055 in COV^+^ controls (*n* = 3) for (**a**,**b**), respectively, for VGSC activators. Net absorbance values were 1.170 ± 0.046 and 1.113 ± 0.067 in COV^−^ controls (*n* = 3), and 0.136 ± 0.051 and 0.145 ± 0.060 in COV^+^ controls (*n* = 3) for (**c**,**d**), respectively.

In the presence of the two VGSC activators, net absorbance data of all nine concentrations obtained in OV^−^ conditions remained close to the ones measured in COV^−^, COV^+^ and RCV controls regardless of the toxin concentrations tested, whereas in OV^+^ conditions, a sigmoidal dose-response curve was obtained for both P-CTX3C and PbTx3 (Figure 7a,b). In the presence of the two VGSC inhibitors, net absorbance data of all nine concentrations obtained in OV^−^ conditions remained close to the ones measured in COV^−^ and RCV controls regardless of the toxin concentrations tested, whereas in OV^+^ conditions, a sigmoidal dose-response curve was obtained for both STX and dc-STX (Figure 7c,d). 

Table 2 details several characteristic parameters of CBA-N2a curves. The EC_50_ values roughly corresponded to twice that of EC_80_ for VGSC activators and to 2.5-fold higher than that of EC_20_ for VGSC inhibitors. Overall, high reproducibility was found for top and bottom absorbances as well as negative Hillslopes with CVs < 5% for P-CTX3C and PbTx3, and CVs < 10% for STX and dc-STX (Table 2). Regarding VGSC activators, CVs were ≈ 19% and between 17% and 21% for EC_80_ and EC_50_ values, respectively. For VGSC inhibitors, CVs of EC_20_ and EC_50_ varied between 10.3% and 17.5% for STX, and between 19.2% and 26.3% for dc-STX. Comparing the potency within VGSC acting toxin family, P-CTX3C was approximately 3300-fold more potent than PbTx3 and STX was approximately 4-fold more potent than dc-STX (Table 2). 

All these findings showed that this newly improved CBA-N2a allowed for specific and sensitive detection of VGSC activators and inhibitors.

Based on these results, quality check (QC) controls were defined as follows:

- The “QCOV^−^” is the quality control that serves to check for the final viability of N2a cells after 19h of additional culture in the absence of O/V treatment and in the presence of VGSC acting toxins.
- The “QCOV^+^” is the quality control that serves to check for the final viability of N2a cells after 19h of additional culture under O/V treatment and in the presence of VGSC acting toxins.

The QC controls are used to verify the specific action of VGSC acting toxins (Figure S2) and their concentration is selected at the EC_50_ of a given standard toxin.

#### 2.4.3. Composite Toxicity Estimation of VGSC Activators in Fish Samples

Four fish samples were analyzed: two steephead parrot-fish (*Chlorurus microrhinos*, Scaridae, Cmic02 and Cmic19) and two honeycomb groupers (*Epinephelus merra*, Serranidae, Emer05 and Emer13). To evaluate the composite CTX-like toxicity, a set of standards must be run in parallel with samples in order to calibrate an experiment properly. For this purpose, P-CTX3C standard was tested in parallel with fish matrix, in the absence vs. under non-destructive O/V treatment at 85.7/8.57 µM (final concentrations), as the EC_50_ of standards are further needed to infer toxin content in biological samples. 

A high repeatability and reproducibility of viability data were obtained for RCV, COV^−^, COV^+^ controls as well as QCOV^−^ (Table 3), with CVs < 7%, whereas a lower reproducibility was observed for QCOV^+^ absorbance data with a CV of 17.3% inherent to the cytotoxicity of PbTx3 at EC_50_ (Section 2.4.2).

Moreover, statistical analyses by means of the Wilcoxon test showed no significant difference between RCV control and COV^+^, and between QCOV^−^ and COV^−^ for both intra- and inter-assay (Table S1). Conversely, significant differences were observed between QCOV^+^ and all other controls. In addition, the comparison between intra- and inter-assay values showed no significant differences for COV^+^ values, nor between RCV control and COV^+^ values showing that final viability was similar to initial viability under non destructive O/V treatment (Table S1). 

The dose-response curves thus obtained for P-CTX3C (data not shown) allowed to establish another set of EC_80_ and EC_50_ (Table 4) which were compared to those of Table 2 (Section 2.4.2). Statistical comparison by means of the Wilcoxon test showed no significant differences between EC_80_ and EC_50_ values obtained under two distinct O/V treatments, i.e., 100/10 and 85.7/8.57 µM with *p*-values > 0.4 (Table S2). 

Further, the limit of detection (LOD) and the limit of quantification (LOQ) of the CTX-like toxicity in fish were determined using the MCE of 10,000 pg dry extract/µL and EC_80_ and EC_50_ values obtained for P-CTX3C exclusively run in parallel with fish samples under 85.7/8.57 µM OV treatment. The LOD and LOQ showed high repeatability with CVs < 9.5% (intra-assay), whereas a lower reproducibility was noticed with CVs > 23.9% (Table 5). The Wilcoxon test also confirmed no significant differences between intra- and inter-assay data with *p*-values of 0.7 and 0.4 for LOD and LOQ, respectively. Hence, a mean LOD value was established at 0.089 ± 0.017 ng/mg of dry extract for P-CTX3C, with LOQ values about twice that of LOD.

Based on the “dry extract weight/fresh weight” (DEW/FW) ratio characterizing each fish sample (Section 5.2), the LOD and LOQ of P-CTX3C in fish flesh were determined and expressed in ng of P-CTX3C eq/g of fish flesh (Table 5) for further comparison with the EFSA and US Food and Drug Administration (FDA) advisory level. Although LOD and LOQ of the CTX-like toxicity varied from one fish to another (Table 5) due to differences in DEW/FW ratios, the Wilcoxon test showed no significant differences between intra- and inter-assay data regardless of the fish sample and P-CTX3C used as reference (Table S3). Hence, the mean LOD values were established at 0.031 ± 0.008 (CV = 25.4%) and LOQ = 0.064 ± 0.016 (CV = 24.3%) ng P-CTX-3C eq/g of fish flesh.

**Figure 8:**
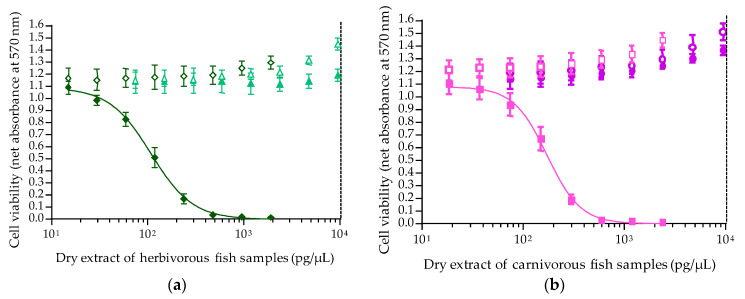
Dose-response curves displayed by N2a cells when exposed to increasing concentrations of fish dry extracts (LF90/10) of two herbivorous fishes (**a**) and two carnivorous fishes (**b**) after 19 h exposure time in OV^−^ (open symbols) and OV^+^ conditions 85.7/8.57 µM (solid symbols): Cmic02 (∆/▲), Cmic19 (◊/♦), Emer13 (○/●) and Emer05 (□/■). Data represent the mean ± SD of one microplate in three independent experiments (N2a cells at 795, 797 and 798 P), with each concentration run in triplicate. The dotted vertical line corresponds to the maximum concentration of dry extract (MCE = 10,000 pg/µL) for matrix interferences.

Application of the CBA-N2a to the detection of VGSC activators in four fish samples gave two distinct patterns as observed for VGSC activators (Figure S1). When exposed to increasing concentrations of fish extracts (Figure 8a,b), no cytotoxic effects were observed in OV^−^ and OV^+^ conditions for Cmic02 and Emer13. Conversely, sigmoidal dose-response curves with a negative slope were obtained for Cmic19 and Emer05 fish samples in OV^+^ conditions (Figure 8a,b), whose ciguatoxicity was previously characterized by fluorescent receptor binding assay (fRBA) and/or LC-MS/MS analyses (Section 5.1.3). 

The sigmoidal dose-response curves thus obtained for Cmic19 and Emer05 were fitted according to the four parameter logistic regression (4PL) model showing high repeatability and reproducibility for top absorbance and Hillslope, with CVs < 10% (Table 6). Absorbance values close to 0 were also consistently obtained at the highest concentration of fish extract tested (Table 6).

The mean EC_50_ and EC_80_ values determined in pg of dry extract/µL for Cmic19 and Emer05 also showed a high repeatability with CVs between 5% and 11.2%, respectively, whereas a lower reproducibility of these values was noticed with CVs between 12% and 21.6%, respectively (Table 6). The Wilcoxon test also confirmed no significant differences between intra- and inter-assay data with *p*-values of 0.7 and 0.2 for Emer05 and Cmic19, respectively. Hence, mean EC_80_ values were established at 63.6 ± 12.4 and 108.5 ± 13.7 pg/µL for Cmic19 and Emer05, respectively, with mean EC_50_ values 1.6 and 1.9 higher than that of EC_80_ for Emer05 and Cmic19, respectively. Based on EC_50_ values, Cmic19 dry extract was 1.5-fold more potent than Emer05 dry extract (Table 6). 

In addition, the toxin contents in fish flesh were also estimated using the EC_50_ values determined for toxin standard and fish extract (Table 7). 

Overall, CTX-like composite toxicity data showed high repeatability and reproducibility with CVs < 13% (Table 7). The Wilcoxon test showed no significant differences between intra- and inter-assays for toxin contents with *p*-values of 0.3 and 0.6 for Emer05 and Cmic19, respectively. Hence, the mean toxin content in Cmic19 and Emer05 was estimated at 6.66 ± 0.68 and 3.31 ± 0.35 ng P-CTX-3C eq/g fish flesh, respectively, indicating Cmic19 was twice as toxic as Emer05.

## 3. Discussion

Having sensitive and specific detection methods available to protect consumers against poisoning risks due to seafood contaminated with marine biotoxins is a key component of food security monitoring programs worldwide [1,5]. However, in the absence of duly validated reference methods, many marine biotoxins are still unregulated [5]. This is the case for instance for CTXs, neurotoxins active on VGSCs that are implicated in ciguatera poisoning [5]. Among the many detection tests currently available for the detection of this group of compounds and, more widely, of toxins acting on voltage-gated sodium channels [8,99], the neuroblastoma cell-based assay or CBA-N2a appears as a very sensitive, specific functional assay [3,12]. However, the numerous protocols found in the literature highlight the lack of a consensus standardized protocol for CBA-N2a. It should be noted that the same observation applies to other analytical methods used for CTXs determination in biological samples such as LC-MS/MS and references therein, [99,100,101]. In this context, the present work aimed at revisiting several key parameters of the CBA-N2a as a step towards the standardized and reliable detection of two groups of toxins frequently involved in toxic outbreaks, namely VGSC activators (e.g., CTXs, PbTxs) and inhibitors (e.g., STX, dc-STX).

### 3.1. Revisit of the CBA-N2a

Overall, six key parameters of the CBA-N2a were revisited.

The first parameter was the cell layer density reached in microplate wells following a 22 to 26 h growth period at 37 °C, as assessed by measuring the absorbance values of the cell layer. The growth curve of the N2a cell line used in this study was established, demonstrating that the maximum number of cells that can be supported in 0.32 cm^2^ wells could not exceed ≈ 100,000 cells. Our data also showed that seeding microplates at 50,000 cells/well allowed reaching this high cell density after ≈ 22 h of culture, with 100% confluence of cell layer in wells, consistent with results obtained in previous studies [59,77]. Obtaining maximum confluence with cells in late log growth phase ensures optimal absorbance data [23]. In the literature, however, a cell layer confluence > 90% was reportedly reached using different cell seeding densities, e.g., 10,000 cells/well [63], 30,000 cells/well [64,73] and 100,000 cells/well [19,20,47,48]. Some authors reported higher cell seeding densities of 250,000 cells/well [57,71], but such assay conditions do not appear physically or physiologically relevant. Actually, our results suggest that it is not the cell seeding density in itself that matters when seeking to achieve an optimal implementation of cell layer in culture microplates, and that this parameter should be adjusted to both the growth rate of the N2a cell line and the culture conditions used in the different laboratories. Therefore, growth experiments should be conducted in each laboratory in order to characterize the growth pattern of the cell line(s) in use.

The second parameter examined in this study was the MTT incubation time for cell viability assessment [19]. Here, the MTT assay was standardized using an optimal wavelength of 570 nm and an incubation time of 45 min, allowing to obtain reproducible absorbance values ≥ 1 when a maximum density of adherent cells is reached in wells after 26 h of growth, i.e., ≈ 100,000 cells/well. In all further experiments, this condition which serves as an indicator of the good health conditions of N2a cells before exposure to toxic extracts was referred to as the “initial cell viability”, and was assessed by implementing a RCV control microplate. Moreover, setting the initial cell viability to an absorbance value ≥ 1 (=baseline viability) allowed us to establish a full sigmoidal dose-response curve derived from testing an eight points serial dilution of the toxin standard/sample. Any subsequent drop in cell viability following O/V treatment and toxin exposure, could then be most likely attributed to the specific effect of toxin standard/biological sample, and not from non optimal culture conditions or oversensitivity of cells. 

Regarding the selection of a wavelength at 570 nm, our results are in agreement with previous work showing that the peak absorbance of MTT formazan precipitate is classically observed at 562 nm with shoulders at 512 and 587 nm [29]. Wavelengths within this range have been used in a number of studies, [36,42,50,64,67,87,90], and were generally associated with MTT incubation time around 30 min. However, wavelengths outside this range were also tested, resulting in a weaker optical signal [29] consistent with the fact that the use of lower [45,76,84,85] or higher wavelengths [57,61,79,93] often required longer MTT incubation times (>60 min). 

The third parameter analyzed was the final viability of N2a cells under OV^−^ conditions, following an additional culture period of 19 h, overnight. Indeed, the N2a growth curve previously established showed that a loss in N2a cell viability is likely to occur at this stage when a high cell density is reached. To prevent this, a complete renewal of the used growth medium with a 2% FBS culture medium was found sufficient to stabilize cell viability throughout the CBA-N2a, as evidenced by absorbance data that were consistently found ≥1 in COV^−^ control wells. This operation appears all the more relevant since MTT assay does not actually measure the number of viable cells or their growth, but the activity of an integrated set of enzyme linked to cell metabolism [24]. The necessity of this medium renewal has been previously highlighted in many studies [34,49,51,56,71,79,80,87,93] although the benefits of such a procedure were never clearly explained. 

The fourth parameter revisited was the concentrations of Ouabain and Veratridine applied in O/V treatment. In this study, we were able to determine two distinct ranges of O/V concentrations to use depending on the group of toxins targeted in the CBA-N2a, i.e., VGSC activators and inhibitors. For the specific detection of VGSC activators in which a loss in cell viability is sought, non-destructive concentrations of O/V with respect to N2a cell viability ranging from 80/8 and 100/10 µM are recommended in order to maintain a final cell viability in COV^+^ control close to the initial cell viability of RCV control (absorbance values ≥ 1). Conversely, for the specific detection of VGSC inhibitors in which a restoration of cell viability is sought, destructive concentrations of O/V ranging from 270/27 to 300/30 µM should be used in order to induce a residual cell viability in COV^+^ control corresponding to approximately 10% (absorbance value of ≈ 0.1) of the initial cell viability measured in the RCV control. Moreover, the ranges of these two types of O/V treatments seem to induce constant effects on N2a cells although high passages were used and that these latter were distant from each other up to 420 passages. However, passage specific effects were not tested directly in this study, and may be the focus of future work. These findings are currently verified on the N2a cell line used in our laboratory at lower number of cell passages. The 500/50 µM O/V treatment originally proposed by Jellett [17] to detect STX was in fact chosen to induce “a lysis of many of the cells in 24 h incubation time”. Such characteristics reinforce the idea that the CBA-N2a is a bioassay that is function-specific rather than toxin-specific [102].

The use of 83/17 µM [50] or 100/10 µM [83] in O/V treatment has been reported in the literature for the detection of PbTxs, although a 10–20% reduction in cell viability was observed. Similarly, a 100/10 µM O/V treatment is often selected for the detection of CTXs in *Gambierdiscus* spp. extracts [56,65,79,80,93] and fish samples [54,55,57,71,74,80], although in many of these studies no information on whether a renewal of the growth medium was applied prior to O/V treatment or on the efficiency of O/V treatment was provided. Consistent with our results is the report of the use of a 300/30 µM O/V treatment in previous studies aiming at detecting STX, which resulted in an 80% reduction in cell viability [83,94]. Additionally, previous studies showed a reduction of cell viability of 40% and up to 69–92% without and following the renewal of the growth medium, respectively, under O/V treatment at 500/50 µM [34,96]. In the present study, an O/V treatment at 300/30 µM was sufficient to induce a 100% reduction in cell viability, suggesting that the renewal of the medium was beneficial to cell physiology, thus allowing to optimize the effect of O/V treatment. However, the extent to which reduction of FBS in the culture medium from 5% to 2% following medium renewal might have rendered the cells more sensitive to toxins remains to be tested in follow-up experiments. The direct addition of O/V treatment in old culture medium following cell layer implementation in wells [19] should be avoided to ensure appropriate preservation of cell viability and further O/V treatment efficiency. 

The fifth parameter examined was the concentration of solvents used to resuspend toxin standard solutions or dry extracts. This study showed that, for MeOH and DMSO, the highest final concentrations should be 0.5% to avoid non-specific cytotoxic effects on N2a cell viability, which is ensured by a dilution of at least 1:200 of the sample stock solutions. These possible solvent interferences were addressed in various ways in previous studies, including working at a final solvent concentration of 0.25–0.3% [11,87], performing an additional evaporation step before re-dissolution of toxins or biological samples in RPMI medium [54,74,81,91,103], or adding 1% MeOH in controls wells to identify any cytotoxic effects [64,73] or 5% MeOH known to induce no more than 20% of cytotoxicity on cell lines [53].

The sixth and last parameter was the maximum amount of extract to expose to avoid potential matrix effects (MCE) as compounds that often co-extract with the analyte(s) of interest can induce unspecific effects on N2a cell viability [81]. In this study, this value was determined at 10,000 pg of dry extract/mL based on the LF90/10 dry extract weight (DEW) instead of the fresh tissue equivalent (FW) used to prepare these extracts, given the significant differences often observed in the DEW/FW ratio of biological samples. Interestingly, only one previous study has used this rationale and has calculated the MCE based on lipid extracts [84]. It should be noted that MCE values are likely to vary according to the nature of tissue analyzed (e.g., flesh, viscera, fin, etc.), the different trends in lipid and water contents in tissue when sampled at different stages of the life history of a given fish species [104]. Moreover, MCE also depends on the extraction protocols and extraction efficiency [46,57,73,105], as shown for CTXs for which no reference consensus extraction protocol is available as yet [105]. Thus, until a standardized universal protocol is made available, researchers conducting similar studies should necessarily perform their own matrix assessments before applying CBA-N2a.

### 3.2. Performance of the Revisited CBA-N2a

The accuracy of the data provided by this revisited CBA-N2a, relies, in part, on the availability of five viability controls, i.e., RCV, COV^−^, COV^+^, QCOV^−^ and QCOV^+^ that were established throughout the various stages of CBA-N2a in order to verify: (i) the initial (RCV control) and final viability of N2a cells (COV^−^ controls), (ii) the efficiency of O/V treatment (COV^+^ controls) and (iii) the detection of the specific mode of action of VGSC activators vs. inhibitors (QCOV^−^ and QCOV^+^ controls). If the use of percentages is recurrent in most studies to express cell viability results [7,8,32,33,36,37,43,46,53,59,62,63,66,67,68,70,73,74,76,81,83,85,87,88,90,91,95,97,103,106,107,108,109,110,111,112], the originality of our revisited CBA-N2a is to recommend the use of absorbance data by establishing a RCV control that characterizes the baseline viability of N2a cell layer prior to O/V treatment and toxin exposure. This RCV control serves as a reference from which all other viability controls as well as viability data of standard and samples depend. This RCV control could be considered as a common reference across all laboratories employing this method. 

As a result of this comprehensive revisit of the CBA-N2a, a practical guide with a step-by-step protocol was defined (Supplementary Materials), that is accessible to both supervised beginners and experienced users. It also provides useful decision limits when interpreting CBA-N2a data in the framework of routine CBA-N2a based monitoring programs.

In this study, the sensitivity of the CBA-N2a was characterized by EC_50_ values found for P-CTX3C at 1.7 ± 0.35 pg/mL, consistent with previously published values of 1.3 ± 0.06 pg/mL [46], 1.66 ± 0.16 pg/mL [64,73] and 1.44 ± 0.70 pg/mL [77,78]. However, quite different values can also be found in the literature, e.g., 0.57 ± 0.11 pg/mL [90], 0.914 ± 0.127 pg/mL [36] and 3.10 ± 0.76 pg/mL [59]. These discrepancies can be attributed to different experimental conditions including different concentrations of O/V treatment [73] or the use of MeOH as a sample vehicle for higher sensitivity [90]. The EC_50_ found for PbTx3 was 5.8 ± 0.9 ng/mL, which is lower than the previously published value of 65.60 ± 23 ng/mL [37].

When comparing the mean EC_50_ values of P-CTX3C to those obtained via other functional assays, they were found 350-fold lower than those estimated via the radioactive receptor binding assay (rRBA), i.e., 0.62± 0.16 ng/mL [113] and 0.61 ± 0.01 ng/mL [46], and the fluorescent RBA, i.e., 0.66 ± 0.16 ng/mL [73], respectively. For PbTx3, EC_50_ values were 2-fold higher than those derived from rRBA experiments (2.77 ± 1.09 ng/mL [37] and 2.06 ± 0.16 ng/mL [113]). These discrepancies observed between functional assays can be explained by the fact that the activity of these polyether toxins depends not only on their mode of action, and their affinity for specific sodium channel isoforms [114], but also on their efficacy on other ion channels and references therein, [37,115,116,117]. As for the detection of STX and dc-STX, the comparison of CBA-N2a results obtained in this study with other functional assays was not possible since previous studies used different units. 

For VGSC inhibitors, STX and dc-STX displayed typical dose-response curves with positive Hillslope in the presence of lethal 270/27 µM OV treatment giving EC_50_ values of 3 ± 0.5 and 15.8 ± 3 ng/mL for STX and dc-STX, respectively. At the EC_20_, this revisited CBA-N2a could specifically detect inhibition of VGSCs at 1 and 5 ng/mL for STX and dc-STX, respectively. This result is consistent with a detection limit established at 2 ng/mL for STXs by Manger et al. [19].

This improved CBA-N2a was further applied to the detection of CTX-like toxicity in fish matrix, giving mean LOD and LOQ values of 0.031 ± 0.008 and 0.064 ± 0.016 ng P-CTX3C eq/g fish flesh, respectively. In order to compare these results with those obtained via analytical techniques such as LC-MS/MS data, this LOD value was further expressed in P-CTX1B equivalent, taking into account a toxicity equivalent factor (TEF) of 0.2 for P-CTX3C [118]. A LOD value of 0.0062 ng P-CTX1B eq/g. was found below the recommended threshold of 0.01 ppb for P-CTX-1B [118,119], indicating that both CBA-N2a and LC-MS/MS share similar level of sensitivity for the detection of P-CTXs [61,100,120,121,122]. However, chromatographic analyses remain unavoidable for the formal characterization of the different structural analogs (toxin congeners) present in biological samples.

Based on EC_50_ data, the toxin content in four fish samples of known ciguatoxic status was further checked via the CBA-N2a. Only two fish specimens, namely *Chlorurus microrhinos* (Cmic19) and *Epinephelus merra* (Emer05) were found to contain 6.66 ± 0.68 and 3.31 ± 0.35 ng P-CTX-3C eq/g fish flesh, respectively, corresponding to ≈ 1.34 and 0.65 ng P-CTX1B eq/g, which are 134- and 65- fold above the advisory level recommended by both the US FDA and EFSA [118,119]. The composite cytotoxicity detected in the Cmic19 *Chlorurus microrhinos* is in agreement with fRBA and LC-MS/MS analyses, which confirmed the presence of six distinct P-CTX congeners in this fish [120]. Interestingly, these toxicity data also confirmed that the overall toxicity of herbivorous fish (parrot-fish) can sometimes surpass that of a carnivorous fish (grouper), although CTX toxin profiles occurring in individuals with different trophic habits can differ significantly owing to the biotransformation processes that occur along the food chain [120,123,124]. In addition to biotransformation, several other traits can be responsible for the unique CTX toxin profiles of individual fish, including site-specificity, feeding behavior and ontogenetic dietary shifts [61,123,125].

Several intra- and/or inter-assay comparisons of the revisited CBA-N2a were performed to assess the coefficients of variations (CVs) obtained for key parameters of CBA-N2a dose-response curves, and evaluate the repeatability and reproducibility of the method. Results indicate that for both VGSC activators and inhibitors, EC_50_ values showed CVs ranging from 10% to 26%, which are considered as acceptable values for functional tests. These results are congruent with previous studies showing 3.5 to 25% and 5 to 24.7% of CBA-N2a variability for intra and inter-assay, respectively [46,80,93,126]. A variability of up to 30% is generally admitted for other functional tests [127,128,129]. Additionally, no significant differences were found for EC_50_ and EC_80_ values of P-CTX3C and PbTx3 standards obtained from two non destructive O/V treatments run at distinct cell passages. Using different OV concentrations selected in the range of 80/8–100/10 µM and different numbers of cell passage seem to not impact the values of EC_50_ and EC_80_ in our study. For LOD and LOQ values, CVs below 29% were obtained vs. CV of 13% for composite toxicity estimates in the two ciguatoxic fishes.

### 3.3. Advantages of the Modified Protocol 

An advantage of the CBA-N2a is necessary material and reagents, as well as basic laboratory equipments are readily (commercially) available.

Several opportunities to reduce reagents were identified at different steps of the test, e.g., using 5% instead of 10% FBS culture medium for the seeding of N2a cells, renewing culture medium with only 2% FBS growth medium as opposed to 5% or 10% FBS commonly used in many studies [34,49,51,56,71,79,80,87,93], or working at lower O/V concentrations for O/V treatment than those originally proposed [83]. Moreover, the direct addition of O/V solution into the renewed growth medium also contributes to a better homogeneity of O/V deposit for increased repeatability and reproducibility, and a reduction of variability sources by limiting the number of pipetting. Finally, this revisited protocol allowed the use of reduced amounts of expensive toxin standards with e.g., only 80 pg of P-CTX3C toxin standard required per microplate to establish a full dose-response curve.

Another advantage of this improved test is its high modularity with respect to the specific detection of a wide range of toxins acting on VGSCs, i.e., both VGSC activators and inhibitors, thanks to the definition of O/V treatment conditions allowing the visualization of a drop in cell viability vs. cell layer restoration, respectively. This protocol also offers the possibility to assay as many as 15 microplates in one experiment (in addition to the one used for RCV control) that can be used either for the qualitative screening of 120 distinct samples at a single concentration (Figures S3 and S4), or the composite toxicity analyses of 14 samples and one toxin standard tested at eight distinct concentrations (Figures S5 and S6), depending on the laboratory research goals. For instance, the high throughput screening approach would be more suitable in risk monitoring programs aiming at the random testing of a high number of wild fish specimens, e.g., to confirm the bioaccumulation of CTXs in the marine fauna and/or the emergence of CP in novel areas. The composite toxicity estimation would be preferred when confirmatory analyses in fish products are required, e.g., to confirm the diagnosis of CP in patients, or help link the CTX content in fish to the symptoms and/or severity of the poisoning. Alternatively, using a two-tiered approach in monitoring programs is also feasible, i.e., perform rapid screening tests on a whole batch of fish specimens, and then conduct composite toxicity estimations only on those specimens found suspect or positive.

### 3.4. Limitations and Gaps of the Present Study

Despite the significant improvements described in the present study, the effects of several parameters that were not tested here remain to be evaluated prior to the standardization and widespread use of this method by different laboratories. For example, parameters inherent to slight differences in N2a cell lines across laboratories due e.g., to mycoplasma infection of cell cultures [130,131], cell passage numbers [132,133], desensitization treatment [134] or the use of different mammalian cell lines [53,66,83,135] should be considered in follow-up studies, as they are likely to affect cell growth rates and cell responses to toxins in this functional assay. Additional studies about the effects of reagents, media exchange, media renewal, etc. are also needed. Likewise, the inclusion of a limited number of fish in the matrix interference study presently limits the application of the method for general detection of VGSC toxins in other matrices (e.g., screening of PbTxs and STXs in shellfish samples). Another major issue is the current lack of a duly validated standardized extraction protocol for the detection of CTXs [100,105]. To this respect, the MCE value determined here must not be regarded as a universal dose as MCE closely depends not only on the extraction protocol used but also on the nature of the extract being tested [105]. Therefore, laboratories would need to perform their own matrix assessments prior to CBA-N2a studies.

## 4. Conclusions

Today, CBA-N2a is widely used for the detection of CTXs in a variety of biological samples, other than fish, including *Gambierdiscus* cells [46,59,64,65,79,91,93,136], giant clams [59,137], gastropods [77], sea urchins [46,78], lobsters and crabs [61], sharks [103] and even samples derived from passive monitoring sampling devices [138,139,140], highlighting the benefit of incorporating this functional assay into routine ciguatera risk monitoring programs for increased food security of populations worldwide. However, due to the lack of a validated reference detection method, this toxin group is still unregulated. Owing to the good performance of this revisited CBA-N2a, this improved method could reasonably be regarded as a first step towards the implementation of a reference functional detection test, provided its further validation through inter-laboratory studies. To this end, significant progress needs to be achieved in addressing several other issues such as the current shortage of certified standards and reference materials as well as the lack of consensus extraction protocols.

## 5. Materials and Methods

### 5.1. Biological Material

#### 5.1.1. Neuroblastoma Cell Line Culture

Mouse neuroblastoma (N2a) cell line (CCL-131) was purchased from the American Type Culture Collection (ATCC, Manassas, VA, USA) (Table S4). The complete growth medium consisted of RPMI medium 1640 with HEPES and without L-Glutamine, supplemented with 10% fetal bovine serum (FBS), 2 mM GlutaMAX, 1 mM sodium pyruvate, 50 µg/mL streptomycin with 50 units/mL penicillin and 2.5 µg/mL Amphotericin B. This cell line was routinely maintained at 37 °C in a 5% CO_2_ humidified atmosphere by splitting cell culture by 1:10 (every two days) or 1:30 (every three days) by rinsing the cell layer with Dulbecco’s Phosphate Buffered Saline without CaCl_2_ and MgCl_2_ (DPBS-1X), followed by a dissociation step using 0.05% Trypsin-EDTA. The N2a cell line used in this study showed a stabilized growth after 300 P. All experiments presented in this study were done using cell passages ranging from 383 to 810 P.

All reagents are listed in Supplementary Materials (Table S5). N2a cell line was sub-cultured in 25 cm^2^ or 75 cm^2^ tissue culture flasks (Nunc™ Easy-Flask, ThermoFisherScientific, Kamstrupvej, Danemark), while 96-well (0.32 cm^2^/well) flat bottom microplates (Falcon^®^, Corning Brand, New York, NY, USA) were used for CBA-N2a experiments. The list of equipments and materials are presented in Supplementary Materials (Table S6).

#### 5.1.2. Reagents and Toxin Standards

Ouabain octahydrate (O3125), Veratridine (V5754), MTT, DMSO and MeOH are listed in Supplementary Materials (Table S4). Aqueous stock solutions of Ouabain and Veratridine were prepared at 20 mM in pure water and 5 mM in pH2 pure water, respectively.

Four toxin standards were used to characterize the mode of action of two toxin families using the CBA-N2a (Table S4): (i) two VGSC activators for which certified reference material is not available as yet, i.e., Pacific ciguatoxin P-CTX3C obtained from the bank of standards of Institut Louis Malardé [77,78,141] and brevetoxin PbTx3 (ref. L8902) purchased from Latoxan (Valence, France). Stock solutions of P-CTX3C and PbTx3 were prepared in DMSO at 20 ng/mL and 100 µg/mL, respectively. Non volatile DMSO solvent was chosen to ensure stable toxin concentrations over long periods of storage; (ii) two VGSC inhibitors for which certified reference materials were purchased from the National Research Council Canada (NRCC, Halifax, NS, Canada), i.e., saxitoxin STX (ref. CRM-STX-f) supplied at a concentration of 66.3 µM in aqueous hydrochloric acid 3 mM and decarbamoylsaxitoxin dc-STX (ref. CRM-dc-STX-b) supplied at a concentration of 65 µM in aqueous hydrochloric acid 3 mM. Concentrations of the STX and dc-STX standard solutions correspond to 24.7 and 21.4 µg/mL, respectively.

#### 5.1.3. Fish Samples

The four fish samples tested in this study were collected from two ciguatera-endemic areas of French Polynesia, namely Tikehau Island (Tuamotu Archipelago) and Mangareva Island (Gambier Archipelago), and conditioned in the form of fillets kept at −20 °C until further CTX extraction. For some of them, their toxicity was also previously characterized by means of the fluorescent Receptor Binding Assay (fRBA) and/or Liquid Chromatography coupled with Tandem Mass Spectrometry (LC-MS/MS) [73,120]. Table 8 details their geographic origin, species, trophic status, as well as ciguatoxic status.

### 5.2. Extraction Procedure

Fish fillets were carefully homogenized in a blender (Groupe SEB, Lourdes, France) waste disposal unit for 1–2 min [113]. For each fish sample, a portion of 10 g was processed following the protocol described in Darius et al. 2018 [77]. Briefly, a liquid/liquid partition was applied followed by a purification step using Sep-Pak C18 cartridges (360 mg sorbent per cartridge; Waters^®^, Saint-Quentin, France) leading to three distinct liposoluble fractions, i.e., LF70/30, LF90/10 and LF100. Fractions LF90/10 likely to contain CTXs were further dried in a SpeedVac concentrator (ThermoFisherScientific, Waltham, MA, USA) and weighed with a Sartorius Micro balance (model MC 410 S, Sartorius, Göttingen, Germany) with a reading accuracy of 0.1 mg (Table 9). The resulting dry extracts were resuspended in pure methanol at a concentration of 10 mg/mL and kept at −20 °C until further CBA-N2a analysis. Prior to dosing by N2a cells, fish extracts were brought to room temperature. 

### 5.3. Neuroblastoma Cell-Based Assay (CBA-N2a)

#### 5.3.1. Characterization of N2a Cell Growth and Initial Viability

The initial viability of N2a cells is a function of the cell density reached in wells after 26 h growth, their physiological state and the time allocated for the metabolization of MTT by viable cells. 

First, in order to determine the maximum cell density supported by each of the wells (representing a surface of 0.32 cm^2^) of 96-well Falcon^®^ microplates, the growth curve of the N2a cell line used in this study was examined over a period of 4 days. For more convenience, growth experiment was conducted in 25 cm^2^ culture flasks. Twelve culture flasks were seeded with 335,000 cells resuspended in 11 mL 10% FBS culture medium solution, and left to incubate for 22 h as described in Section 5.1.1. Cell densities were then monitored by sacrificing one flask every 6 h starting from 22 to 76 h. The two last flasks were sacrificed at 85 and 100 h, respectively. Cell enumeration was achieved by manual counts (*n* = 10/flask) using KOVA Glasstic slides (Hycor), and the results plotted against time. Cell density data were normalized to a surface of 0.32 cm^2^ and used as a proxy of cell densities in 96-well microplates: e.g., using this rationale, the initial cell density in a 96-well microplate was estimated at 4288 cells per well. 

Second, the initial viability of N2a cells was examined under two different % FBS (5% and 10% in culture medium) and six different MTT incubation times (15–65 min). Cell numbers before seeding were determined from *n* = 5 counts performed on an aliquot of the cell stock suspension and apply for all further experiments. To this end, twelve microplates (six microplates per % FBS) were seeded with 200 µL of ten different cell seeding densities ranging from 10,000 to 100,000 cells/well (*n* = 6 wells per cell density). After 26 h growth, cell viability was assessed via the MTT assay conducted by sacrificing one microplate at 15 min and the remaining every 10 min. MTT and DMSO conditions were used as originally described [19]. The MTT protocol followed the one described in Darius et al. [77]. Briefly, after removing the culture medium from the microplate, the 60 inner wells were filled with 60 µL of MTT solution at 0.83 mg/mL prepared in 2% FBS culture medium. After MTT incubation at 37 °C in a 5% CO_2_ incubator, the wells were emptied and the 60 inner wells and 12 outer wells (rows 1 and 12) were filled with 100 µL of DMSO. Following cell layer lysis with DMSO and manual homogenization, absorbance data were measured at 570 nm using an iMark^™^ microplate reader (Biorad, Marnes la Coquette, France). All viability data were expressed in absorbance data.

#### 5.3.2. Characterization of N2a Cell Final Viability in the Absence and under O/V Treatment

Ouabain and Veratridine (O/V) treatment is required for the successful detection by CBA-N2a of toxins active on VGSCs. The N2a cell final viability, i.e., in the absence of O/V treatment (OV^−^ conditions) or following O/V treatment (OV^+^ conditions) was characterized as follows. 

To characterize the final viability of N2a cells in OV^−^ conditions, six microplates were seeded with 200 µL of a 5% FBS culture medium at ten different cell densities ranging from 10,000 to 100,000 cells/well (*n* = 6 wells per cell density) and left to grow for 26 h. One microplate, defined as the RCV control, was used to check the initial viability of N2a cells after 26 h growth and measured by MTT assay, while the five remaining microplates were treated as follows: culture medium was discarded under sterile conditions by overturning, and wells were dried by tapping each microplate on an ethanol-sterilized absorbent paper to remove any residual liquid. The 60 inner wells of the five microplates then received 200 µL/well of growth medium supplemented with 1%, 2%, 3%, 4% or 5% FBS, respectively, while the peripheral wells received the same volume of sterile distilled water. Culture microplates were further incubated overnight (Section 5.1.1) for an additional 19 h. Cell final viability was assessed using the MTT assay after 45 min incubation with MTT. 

To characterize the final viability of N2a cells in OV^+^ conditions, two microplates were seeded with 200 µL/well of a 5% FBS culture medium at an initial cell density of 50,000 ± 10,000 cells/well, and left to grow for 26 h. After cell layer settlement, one microplate served as RCV control and measured by MTT assay. For the second one, 20 mM (O) and 5 mM (V) solutions were first diluted in 2% FBS culture medium to obtain a 360/36 µM O/V stock solution, which was further used to prepare serial dilutions ranging from 340/34 to 20/2 µM (with a decrease of 20/2 µM between each dilution). After removal of the used culture medium as previously described, each of the 60 inner wells received 200 µL/well of culture medium at nineteen distinct O/V concentrations ranging from 0/0 to 360/36 µM (*n* = 3 wells per O/V treatment conditions, except for 0/0 µM condition for which *n* = 6). Peripheral wells received the same volume of sterile distilled water. Culture plates were then incubated overnight for 19 h. Cell final viability was assessed using the MTT assay after 45 min incubation with MTT. Five independent experiments were performed.

#### 5.3.3. Characterization of the Unspecific Effects of Solvent and Dry Extract on N2a Cell Viability

The effects on N2a cell viability of two solvents commonly used to resuspend toxin standards and dry extracts, i.e., MeOH and DMSO, were examined in the absence vs. under non-destructive O/V treatment conditions. In this experiment, the 60 inner wells of three 96-well microplates were seeded with 200 µL/well of a 5% FBS culture medium at an initial cell density of 50,000 ± 10,000 cells/well, and left to grow for 26 h. One microplate served as RCV control and was measured by MTT assay while the two remaining ones were treated as follows: after the complete removal of the used culture medium as previously described, the 30 inner wells on the upper half of the microplate received 200 µL/well of a 2% FBS culture medium (OV^−^ conditions), while the 30 inner wells on the bottom half of the microplate received 200 µL/well of a 105/10.5 µM O/V treatment (OV^+^ conditions). In parallel, 100 µL of eight-points serial dilution at 1:2 of each solvent were prepared in the same culture medium using a U-bottom 96-well microtiter plate. Then, 10 µL of each solvent dilution were directly added in triplicate wells and tested under OV^−^ conditions versus OV^+^ conditions (100/10 µM final concentrations). Hence, the final concentrations tested ranged from 0.037 to 4.762%. Addition of 10 µL of 2% FBS culture medium in triplicate wells under OV^−^ and OV^+^ conditions (COV^−^ and COV^+^ controls, respectively), allowed to check final viability in solvent-free growth medium. Peripheral wells received the same volume of sterile distilled water. Culture plates were left to incubate overnight for 19 h, and the cell final viability determined using the MTT assay after 45 min incubation with MTT.

In order to determine the MCE of the four fish samples, increasing concentrations of fish extracts were tested in OV^−^ conditions only, as no activity on VGSCs is expected to occur in this condition of treatment [81]. In most CBA-N2a studies however, these concentrations are established based on the fresh tissue weight of biological samples. Here, the tested concentrations were estimated based on the DEW instead, since dry extracts interact directly on cell layers and substantial differences in DEWs are often observed between extracts prepared from a same amount of tissue sample. In order to determine the MCE of the four fish samples used in this study, the 60 inner wells of three 96-well microplates were seeded with 200 µL of a 5% FBS culture medium at an initial cell density of 50,000 ± 10,000 cells/well, then left to grow for 26 h. One microplate served as RCV control and was measured by MTT assay, while the two remaining ones were treated as follows: first, 5% FBS growth medium was renewed by addition of 200 µL of a 2% FBS culture medium in wells. In parallel, a 1:10 dilution of the LF90/10 dry extract of four fish samples was prepared in 2% FBS culture medium using a U-bottom 96-well microtiter plate, followed by a nine-points serial 1:2 dilution (v = 100 µL per concentration). Then, 10 µL of each concentration was directly added in triplicate wells, leading to final dry extract concentrations that ranged from 186 to 47,619 pg/µL, i.e., 0.517 to 132.3 µg fish flesh equivalent/µL for Cmic02, 0.433 to 110.7 µg/µL for Cmic19, 0.600 to 153.6 µg/µL for Emer05 and 0.689 to 176.3 µg/µL for Emer13. Two fish samples were tested per plate. Controls wells (COV^−^) were also established by addition of 10 µL of 2% FBS culture medium in the absence of dry extract. Peripheral wells received the same volume of sterile distilled water. Culture plates were left to incubate overnight for 19 h, and the cell final viability determined using the MTT assay after 45 min incubation with MTT. Three independent experiments were performed.

#### 5.3.4. Detection of VGSCs Activators and Inhibitors by CBA-N2a

In this experiment, 60 inner wells of five 96-well microplates were seeded with 200 µL of a 5% FBS culture medium at an initial cell density of 50,000 ± 10,000 cells/well, and left to grow for 26 h. One microplate served as RCV control and was measured by MTT assay, while the four remaining ones were treated as follows: first, the growth medium was renewed by the addition of 200 µL of 2% FBS culture medium in OV^−^ conditions (upper half of the microplate) versus 200 µL of culture medium in OV^+^ conditions (bottom half). The initial O/V concentrations in wells were 105/10.5 and 284/28.4 µM when detecting VGSC activators and VGSC inhibitors, respectively. Further, a nine-points serial 1:2 dilution of each toxin standard stock solution was prepared in 2% FBS culture medium (v = 100 µL per concentration) using a U-bottom 96-well microtiter plate, then 10 µL of each toxin concentration were directly added in triplicate under OV^−^ and OV^+^ conditions (Section 5.3.3). The final concentrations of toxins tested ranged from 0.074 to 19.048 fg/µL for P-CTX3C, 372 to 95,238 fg/µL for PbTx3, 368 to 94,095 fg/µL for STX and 1592 to 407,619 fg/µL for dc-STX. The final O/V concentrations in wells were 100/10 and 270/27 µM when detecting VGSC activators and VGSC inhibitors, respectively. Appropriate controls in both conditions of O/V treatment, COV^−^ and COV^+^, were established to verify the cell layer viability and the effect of O/V treatment, respectively, in the absence of toxins (Figure S7). The resulting full dose-response curves were used to characterize the response typical of a given standard toxin and for further toxin quantification. For each toxin standard, one microplate in three independent experiments was examined for the purpose of inter-assay variability comparison.

#### 5.3.5. Detection of VGSC Activators in Fish Samples by CBA-N2a

This experiment was conducted as previously described in Section 5.3.4, except that the initial concentrations of O/V in wells was set to 90/9 µM. Toxin detection and quantification in biological matrix of unknown varying toxicity require the implementation of additional quality check controls (QC) in OV^−^ and OV^+^ conditions, namely QCOV^−^ and QCOV^+^, to check for the validity of further toxicity results (Figure S2). Practically, in these controls, a known concentration of a VGSC activator is tested, whose effect on N2a cell viability has been pre-established. The QCOV^−^ and QCOV^+^ were established by adding 10 µL of 0.1 µg /mL of PbTx3 in triplicate, to reach a final concentration of 4760 fg/µL of PbTx3 in wells (PbTx3 was preferred to P-CTX3C as it is commercially available). An eight-points serial 1:2 dilution of P-CTX3C and fish dry extracts were prepared (v = 100 µL per concentration) using a U-bottom 96-well microtiter, then 10 µL of each concentration were directly added in triplicate under OV^−^ and OV^+^ conditions (85.7/8.57 µM final concentrations). Hence, the final concentrations of P-CTX3C tested ranged from 0.099 to 12.70 fg/µL and from 74.4 to 9523.8 pg of dry extracts/µL for Cmic02 and Emer13, 14.9 to 1904.8 pg of dry extracts/µL for Cmic19 and 18.6 to 2381 pg of dry extracts/µL for Emer05. The full sigmoidal dose–response curves obtained for each toxin standard and fish samples when tested in parallel in the same experiment were used to determine EC_50_ values and infer toxin content in fish samples (Section 5.3.6). Three microplates in one experiment (*n* = 3) and one microplate in three independent experiments (*n* = 3) were examined for the purpose of intra- and inter-assay variability comparison, respectively. 

Validation of the CBA-N2a results by means of appropriate viability controls are presented in Supplementary Materials (Table S4). 

#### 5.3.6. Absorbance Data and Toxin Analysis

First, for each experimental plate, all raw absorbance data were corrected by deducting the corresponding mean DMSO control absorbance data (*n* = 12) to obtain net absorbance data. Dose-response curves were then established by plotting net absorbance values vs. pure toxins or dry extract concentrations tested, using GraphPad Prism software version 8.1.2 (GraphPad, San Diego, CA, USA) based on a four parameter logistic regression model (4PL) according to the following equation:

Y = Bottom + (Top − Bottom/(1 + 10^((Log(EC_50_) − Log(X)) ∗ Hillslope)) (2)

In which Y is the net absorbance data and X is the concentrations tested (fg/µL), and EC_50_ (fg/µL) represents the effective concentration of dry extract inducing a viability half way (50%) between the basal (Bottom) and the maximal (Top) values of the curve (EC_50_). This parameter is used to establish the toxic potency of each toxin standard.

The EC_80_ and EC_20_ values of toxin standards were inferred from dose-response curves and correspond to the effective concentration of dry extract inducing a viability 80% and 20% between the basal (Bottom) and the maximal (Top) values of the curve, respectively.

The limit of detection (LOD) and quantification (LOQ) of the CTX-like toxicity in fish samples were determined according to the following equations:

LOD = (EC_80_/MCE) (3)

LOQ = (EC_50_/MCE) (4)

where EC_80_ and EC_50_ are the values obtained for P-CTX3C toxin standard, with values expressed in ng P-CTX3C eq/mg of dry extract.

For more convenience, LOD and LOQ values can be expressed in the same unit as the one used in the advisory level recommended by the EFSA and US FDA. Calculations are based on the fresh weight of flesh tissue extracted (FW) and the corresponding dry extract weight (DEW) (Table 9), and use the following equations:

LOD = (EC_80_/MCE) × (DEW/FW) (5)

LOQ = (EC_50_/MCE) × (DEW/FW) (6)

In which LOD and LOQ of CTXs in biological matrix are expressed in ng P-CTX3C eq/g fish flesh.

In the same way, quantification of the composite toxicity in fish dry extracts (T), expressed in ng P-CTX3C eq/mg, was determined by comparing the EC_50_ values of P-CTX3C and fish dry extracts determined in the same experiment, using the following equation:

T = EC_50_ of P-CTX3C/EC_50_ of dry extract (7)

The composite toxicity in biological samples (Q), expressed in ng P-CTX3C eq/g of fish flesh, is determined using the following equation:

Q = T × (DEW/FW)(8)

#### 5.3.7. Data Analyses

Variabilities of CBA-N2a data were examined using the mean ± SD of three microplates tested in one experiment on the same day (intra-assay comparisons) or the mean ± SD of one microplate tested in three independent experiments (inter-assay comparisons). Statistical analyses were performed by means of the Wilcoxon test with significant differences considered at *p*-values < 0.05, using RStudio software version 1.0.153 Version 1.0.153–© 2009-2017 (RStudio, Inc., Boston, MA, USA).

First, the intra- and inter-assay variabilities of the five viability controls (Table S7), RCV, COV^−^, COV^+^, QCOV^−^ and QCOV^+^ controls were assessed from inter-assay experiments conducted at 100/10 µM run at 662-663-666 cell passages and intra- and inter-assay experiments conducted at 85.7/8.57 µM run at 810 and 795-797-798 cell passages, respectively of CBA-N2a experiments presented in Section 2.4.2 and Section 2.4.3 and Table S1.

Second, the Wilcoxon test was applied to search for variability between EC_80_ and EC_50_ of P-CTX3C and PbTx3 obtained from inter-assay experiments conducted at 100/10 µM and run at 662-663-666 cell passages, and intra- and inter-assay experiments conducted at 85.7/8.57 µM and run at 810 and 795-797-798 cell passages, respectively (Table 4, Table S2).

For intra- and inter assay comparison of LOD and LOQ values of the CTX-like toxicity in fish dry extracts, three different P-CTX3C EC_80_ or EC_50_ values were compared to a unique MCE value, i.e., 10,000 pg/µL applying for all fish samples. Then, three LOD and LOQ values were obtained giving a mean ± SD with *n* = 3 for intra- and inter-assay for all fish samples (Table 5).

For intra- and inter assay comparison of LOD and LOQ values of the CTX-like toxicity in fish flesh, one specific MCE value (converted from the DEW/FW ratio) was obtained for each fish sample. Then, three different P-CTX3C EC_80_ or EC_50_ values were compared to a unique MCE value per fish sample. In the same way, three LOD and LOQ values were obtained for each fish giving a mean ± SD with *n* = 3 for intra- and inter-assays (Table 5). Additionally, the Wilcoxon test was successfully applied to search for possible variability between LOD and LOQ values among the four fish samples (Table S3). 

For intra-assay comparison of toxin content in fish sample, three different P-CTX3C EC_50_ values obtained from *n* = 3 microplates were compared to three different fish EC_50_ values obtained from *n* = 3 microplates of a given fish sample run the same day. Then, cross calculation (three different P-CTX3C EC_50_ values x three different fish EC_50_ values) were done giving nine toxin content values and a mean ± SD with *n* = 9 per fish sample (Table 7). 

For inter-assay comparison of toxin content in fish sample, one P-CTX3C EC_50_ value was compared to one fish EC_50_ value for each fish sample per independent experiment run at different times. Then, three toxin content values were obtained from the three independent experiments giving a mean ± SD with *n* = 3 for each fish sample (Table 7).

## Figures and Tables

**Table 1:** Initial viability of N2a cells as assessed in RCV controls (cell layer ≈ 100,000 cell/well obtained after 26 h of growth).

Variability	Experimental Plates	Absorbance Raw Data (1)	DMSO Control (2)	RCV Control (1)–(2)	CVs (%)
Inter-assay *	3	1.170 ± 0.050	0.043 ± 0.0001	1.127 ± 0.050	4.4

* Data represent the mean ± SD of one microplate in three independent experiments (N2a cells at 662, 663 and 666 P).

**Table 2:** Dose-response curve parameters of cell-based assay (CBA)-N2a when detecting VGSC activators or inhibitors.

Parameters	P-CTX3C **	PbTx3 **	STX **	dc-STX **
Top absorbance	1.149 ± 0.028	1.182 ± 0.060	0.917 ± 0.017	0.990 ± 0.038
Bottom absorbance	−0.017 ± 0.028	0.057 ± 0.013	0.149 ± 0.073	0.130 ± 0.049
Hillslope	−1.802 ± 0.074	−2.052 ± 0.102	1.545 ± 0.127	1.290 ± 0.126
EC_80_ * (fg/µL)	0.787 ± 0.155	2950 ± 537	ND	ND
EC_50_ * (fg/µL)	1.700 ± 0.354	5800 ± 926	2982 ± 523	15851 ± 3050
EC_20_ * (fg/µL)	ND	ND	1195 ± 123	5456 ± 1434

* Data represent the mean ± SD of one microplate in three independent experiments (N2a cells at 662, 663 and 666 P). ** OV^+^ conditions: 100/10 and 270/27 µM final concentrations for VGSC activators and inhibitors, respectively. ND = not determined.

**Table 3:** Assessment of five viability controls useful to validate the detection of VGSC activators in fish matrix using the revisited CBA-N2a.

Variability	RCV Control	COV^−^	COV^+^	QCOV^−^	QCOV^+^
Intra-assay *	1.122 ± 0.046	1.264 ± 0.068	1.130 ± 0.029	1.257 ± 0.067	0.448 ± 0.038
Inter-assay **	1.132 ± 0.052	1.165 ± 0.074	1.129 ± 0.078	1.174 ± 0.074	0.409 ± 0.071

* Data represent the mean ± SD of three microplates in one experiment (N2a cells at 810 P). ** Data represent the mean ± SD of one microplate in three independent experiments (N2a cells at 795, 797 and 798 P).

**Table 4:** Variabilities of EC_80_ and EC_50_ for two VGSC activators (P-CTX3C and PbTx3) using the revisited CBA-N2a as assessed under non-destructive O/V treatment conditions.

Variability	O/V Treatment	P-CTX3C		PbTx3	
		EC_80_ (fg/µL)	EC_50_ (fg/µL)	EC_80_ (fg/µL)	EC_50_ (fg/µL)
Inter-assay *	100/10 µM	0.787 ± 0.155	1.700 ± 0.354	2950 ± 538	5800 ± 929
Intra-assay **	85.7/8.57 µM	0.964 ± 0.055	2.004 ± 0.190	3230 ± 270	6241 ± 388
Inter-assay ***	85.7/8.57 µM	0.825 ± 0.236	1.730 ± 0.414	3015 ± 347	5946 ± 788

* Data represent the mean ± SD of one microplate in three independent experiments (N2a cells at 662, 663 and 666 P). ** Data represent the mean ± SD of three microplates in one experiment (N2a cells at 810 P). *** Data represent the mean ± SD of one microplate in three independent experiments (N2a cells at 795, 797 and 798 P).

**Table 5:** Estimation of limit of detection (LOD) and limit of quantification (LOQ) of the CTX-like toxicity in fish flesh using the revisited CBA-N2a under non-destructive O/V treatment at 85.7/8.75 µM.

Variability	Sample ID	ng P-CTX3C eq/mg Dry Extract		ng P-CTX3C eq/g Fish Flesh	
		LOD	LOQ	LOD	LOQ
Intra-assay **	Cmic02	0.096 ± 0.006	0.200 ± 0.019	0.035 ± 0.002	0.072 ± 0.007
	Cmic19			0.041 ± 0.002	0.086 ± 0.008
	Emer05			0.030 ± 0.002	0.062 ± 0.006
	Emer13			0.026 ± 0.001	0.054 ± 0.005
Inter-assay *	Cmic02	0.083 ± 0.024	0.173 ± 0.041	0.030 ± 0.008	0.062 ± 0.015
	Cmic19			0.035 ± 0.010	0.074 ± 0.018
	Emer05			0.026 ± 0.007	0.054 ± 0.013
	Emer13			0.022 ± 0.006	0.047 ± 0.011

* Data represent the mean ± SD of one microplate in three independent experiments (N2a cells at 795, 797 and 798 P). ** Data represent the mean ± SD of three microplates in one experiment (N2a cells at 810 P).

**Table 6:** Dose-response curves parameters of CBA-N2a when detecting VGSC activators in fish samples.

Variability	Sample ID	Top Absorbance	Bottom Absorbance	EC_80_ *** (pg/µL)	EC_50_ *** (pg/µL)	Hillslope
Intra-assay *	Cmic19	1.064 ± 0.007	0.001 ± 0.004	71.2 ± 8	130.6 ± 11	−2.282 ± 0.132
	Emer05	1.110 ± 0.014	0.003 ± 0.004	114.7 ± 10	184.4 ± 10	−2.927 ± 0.271
Inter-assay **	Cmic19	1.084 ± 0.062	−0.003 ± 0.002	55.9 ± 12.1	109.5 ± 21.1	−2.056 ± 0.073
	Emer05	1.081 ± 0.101	0.000 ± 0.002	102.2 ± 15.9	171.3 ± 20.5	−2.675 ± 0.196

* Data represent the mean ± SD of three microplates in one experiment (N2a cells at 810 P). ** Data represent the mean ± SD of one microplate in three independent experiments (N2a cells at 795, 797 and 798 P). *** OV^+^ conditions: 85.7/8.57 µM final concentrations.

**Table 7:** CTX-like composite toxicity estimation in two ciguatoxic fish samples using the revisited CBA-N2a.

Variability	Sample ID	ng P-CTX3C eq/g Fish Flesh	
		Mean ± SD	CVs (%)
Intra-assay *	Cmic19	6.63 ± 0.74	11.2
	Emer05	3.37 ± 0.32	9.5
Inter-assay **	Cmic19	6.76 ± 0.57	8.4
	Emer05	3.10 ± 0.40	13

* Data represent the mean ± SD of three microplates in one experiment (N2a cells at 810 P). ** Data represent the mean ± SD of one microplate in three independent experiments (N2a cells at 795, 797 and 798 P).

**Table 8:** List of fish samples tested for the revisited CBA-N2a.

Genus	Species	Feeding Type	Site	Sample	fRBA **	LC-MS/MS
*Chlorurus*	*microrhinos*	Herbivorous	Tikehau	Cmic02	NT *	NT *
			Mangareva	Cmic19	7.04 [73]	six P-CTXs [120]
*Epinephelus*	*merra*	Carnivorous	Mangareva	Emer05	4.4 ± 1.1 ***	NT *
			Mangareva	Emer13	NT *	NT *

* NT: not tested; ** Fluorescent RBA expressed in ng P-CTX-3C eq/g fish flesh; *** Unpublished data.

**Table 9:** Comparison of LF90/10 dry extract weights prepared from 10 g (fresh weight) of fish samples tested with CBA-N2a.

Sample ID	Size (cm)	Weight (g)	Fresh Weight (FW) (g)	Dry Extract Weight (DEW) (mg)
Cmic02	37	926	10	3.6
Cmic19	45	2230	10	4.3
Emer05	22	185	10	3.1
Emer13	18	80	10	2.7

## Supplementary Materials

The following are available online at https://www.mdpi.com/2072-6651/12/5/281/s1, Figure S1: Examples of pictures obtained from the revisited CBA-N2a, Figure S2: Suggested layouts on a 96-wells microplate at day 2 of the CBA-N2a, Figure S3: Detection (DL) and confirmation (CL) limits adopted in qualitative screening analysis of samples tested for the presence of VGSC activators (under non-destructive O/V treatment), Figure S4: Detection (DL) and confirmation (CL) limits adopted in qualitative screening analysis of samples tested for the presence of VGSC inhibitors (under destructive O/V treatment), Figure S5: Typical dose-response curve expected with a sample positive for VGSC activators, tested at eight distinct concentrations (C1–C8) adjusted to fall below the MCE, Figure S6: Typical dose-response curve expected with a sample positive for VGSC inhibitors, tested at eight distinct concentrations (C1–C8) adjusted to fall below the MCE, Table S1: Comparison between assays of the absorbance data of five viability controls, Table S2: Comparison between assays of EC_50_ and EC_80_ values of P-CTX3C and PbTx3 standards under two non destructive O/V treatments (100/10 µM vs. 85.7/8.57 µM), Table S3: Comparison between assays of LOD and LOQ values (ng P-CTX3C equivalent/g fish flesh) under non-destructive O/V treatment at 85.7/8.57 µM), Table S4: List of biological materials, Table S5: List of reagents, Table S6: List of equipments and materials, Table S7: Expected results for viability controls to enable validation of the assay.
