# Supplementary material for: Revisiting the Neuroblastoma Cell-Based Assay (CBA-N2a) for the Improved Detection of Marine Toxins Active on Voltage Gated Sodium Channels (VGSCs)
Source: Toxins (Basel). 2020 Apr 27;12(5):281. doi: 10.3390/toxins12050281 (PMC7290318; doi:10.3390/toxins12050281)
Supplement: Supplementary file 1 [file toxins-12-00281-s001.pdf]

Supplementary Materials: Revisiting the Neuroblastoma Cell-Based Assay (CBA-N2a) for the Improved Detection of Marine Toxins Active on Voltage Gated Sodium Channels (VGSCs)

Jérôme Viallon, Mireille Chinain and Hélène Taiana Darius

Table of content

1. Illustrations of the revisited CBA-N2a 2
2. Statistical analyses of CBA-N2a data 3
3. Practical guide of the revisited CBA-N2a 7

Figures

Figure S1. Examples of pictures obtained from the revisited CBA-N2a........ 2
Figure S2. Suggested layouts on a 96-wells microplate at day 2 of the CBA-N2a. 11
Figure S3. Detection (DL) and confirmation (CL) limits adopted in qualitative screening analysis of samples tested for the presence of VGSC activators (under non-destructive O/V treatment)........ 14
Figure S4. Detection (DL) and confirmation (CL) limits adopted in qualitative screening analysis of samples tested for the presence of VGSC inhibitors (under destructive O/V treatment). 14
Figure S5. Typical dose-response curve expected with a sample positive for VGSC activators, tested at eight distinct concentrations (C1-C8) adjusted to fall below the MCE. 15
Figure S6. Typical dose-response curve expected with a sample positive for VGSC inhibitors, tested at eight distinct concentrations (C1-C8) adjusted to fall below the MCE. 16

Tables

Table S1. Comparison between assays of the absorbance data of five viability controls. 3
Table S2. Comparison between assays of EC_{50} and EC_{80} values of P-CTX3C and PbTx3 standards under two non destructive O/V treatments (100/10 μM vs. 85.7/8.57 μM). 4
Table S3. Comparison between assays of LOD and LOQ values (ng P-CTX3C equivalent/g fish flesh) under non-destructive O/V treatment at 85.7/8.57 μM). 5
Table S4. List of biological materials 7
Table S5. List of reagents. 7
Table S6. List of equipments and materials 7
Table S7. Expected results for viability controls to enable validation of the assay. 13

1. Illustrations of the revisited CBA-N2a

Here are some illustrations of experimental microplates obtained with the CBA-N2a (Figure S1).

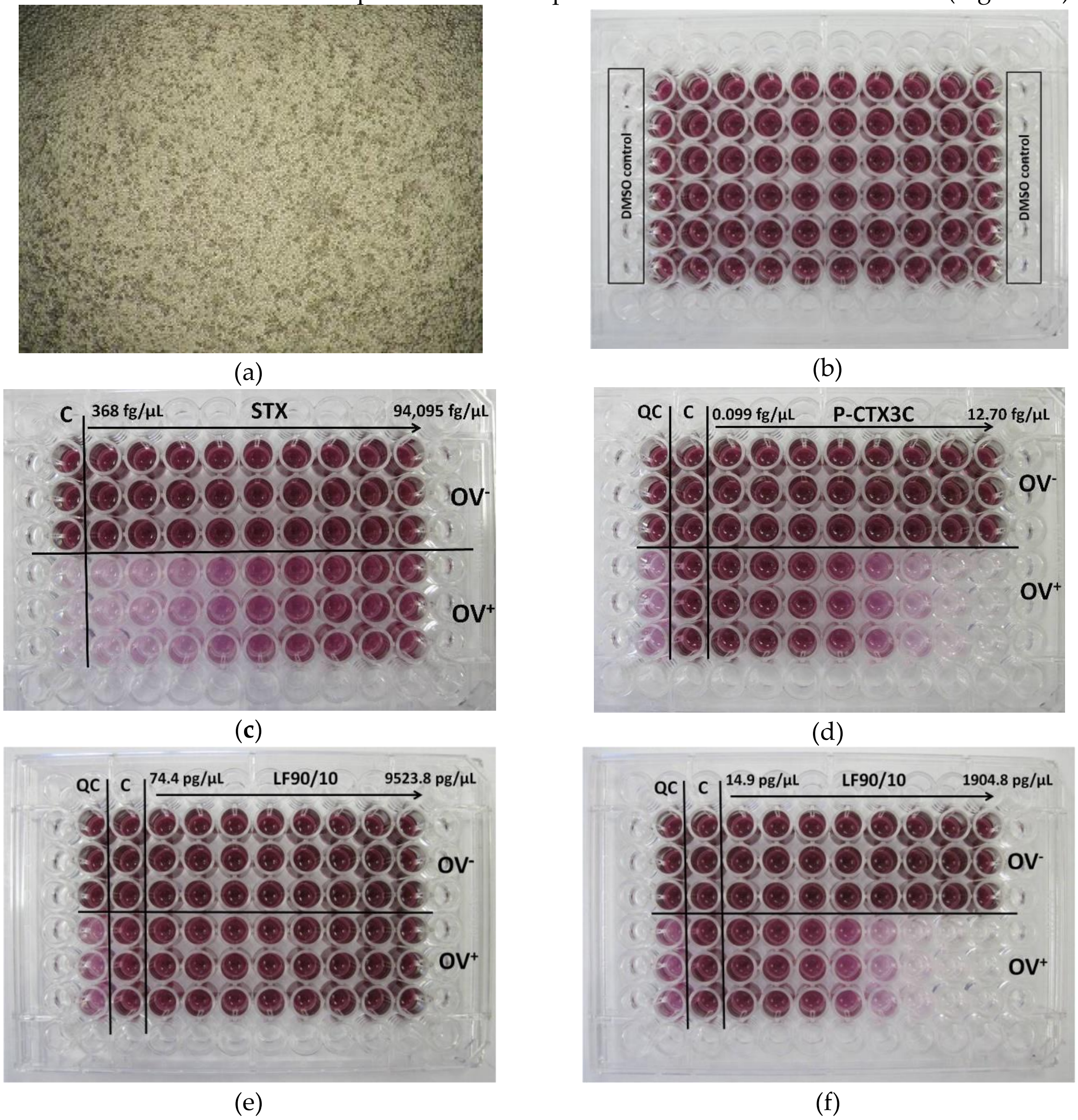

Figure S1. Examples of pictures obtained from the revisited CBA-N2a. (**a**) Cell layer confluence observed in culture wells 26 h after seeding in culture microplates (≈100,000 cells/well and magnification X40); (**b**) RCV control defining the initial N2a cell viability with confluent cell layer of ≈. 100,000 cells/well after 26 h of growth; (**c**) Dose-responses of N2a cells exposed to STX under O/V treatment at 270/27 µM (final concentrations); (**d**) Dose-responses of N2a cells exposed to P-CTX3C under O/V treatment at 85.7/8.57 µM; (**e**) Dose-responses of N2a cells exposed to LF90/10 of non toxic fish sample under O/V treatment at 85.7/8.57 µM; (**f**) Dose-response of N2a cells exposed to LF90/10 of ciguatoxic fish sample under O/V treatment at 85.7/8.57 µM. O/V treatments are given in final concentrations. Nine concentrations for STX and eight concentrations for P-CTX3C and fish samples were tested using a 1:2 serial dilution. Two viability controls (C) without and under O/V treatment (COV^- and COV^+) are shown in column 2 for STX and column 3 for P-CTX3C and fishes. A quality check (QC) using a PbTx3 toxin solution at 4760 fg/µL (final concentration) in both conditions of detection ($QCOV^-$ and $QCOV^+$) is shown in column 2 for P-CTX3C and fishes. The DMSO control used to calculate the net absorbance data is present on both sides of each microplate ($n = 6 \times 2 = 12$).

2. Statistical analyses of CBA-N2a data

Table S1. Comparison between assays of the absorbance data of five viability controls. WILCOXON TEST (*p*-values) (95% confidence level).

		INTRA-ASSAY OV treatment at 85.7/8.57 μM (810P)				
	**CONTROLS**	**RCV control**	**QCOV$^+$**	**QCOV$^-$**	**COV$^+$**	**COV$^-$**
**INTRA-ASSAY** OV treatment at 85.7/8.57 μM (810P*)	**RCV control**		<0.0001	<0.0001	0.4179	<0.0001
	**QCOV$^+$**			<0.0001	<0.0001	<0.0001
	**QCOV$^-$**				<0.0001	0.7060
	**COV$^+$**					<0.0001
	**COV$^-$**					

(**A**)

		INTER-ASSAY OV treatment at 100/10 μM (662-663-666P)				
	**CONTROLS**	**RCV control**	**QCOV$^+$**	**QCOV$^-$**	**COV$^+$**	**COV$^-$**
**INTER-ASSAY** OV treatment at 85.7/8.57 μM (795-797-798P)	**RCV control**		<0.0001	0.0044	0.9656	0.0122
	**QCOV$^+$**			<0.0001	<0.0001	<0.0001
	**QCOV$^-$**				0.0182	0.5772
	**COV$^+$**					0.0531
	**COV$^-$**					

(**B**)

		INTRA-ASSAY OV treatment at 85.7/8.57 μM (810P)				
	**CONTROLS**	**RCV control**	**QCOV$^+$**	**QCOV$^-$**	**COV$^+$**	**COV$^-$**
**INTER-ASSAY** OV treatment at 85.7/8.57 μM (795-797-798P)	**RCV control**	0.0270	<0.0001	<0.0001	0.5053	<0.0001
	**QCOV$^+$**	<0.0001	0.0380	<0.0001	<0.0001	<0.0001
	**QCOV$^-$**	0.0003	<0.0001	<0.0001	0.0132	<0.0001
	**COV$^+$**	0.6005	<0.0001	<0.0001	0.9598	<0.0001
	**COV$^-$**	0.0015	<0.0001	<0.0001	0.0517	<0.0001

(**C**)

A, comparison of the five viability controls between intra- and inter-assay under OV treatment at 85.7/8.57 μM (810P). **B**, comparison of the five viability controls between inter-assays under OV treatment at 85.7/8.57 μM (795-797-798P) and OV treatment at 100/10 μM (662-663-666P). **C**, comparison of the five viability controls between intra-assay under OV treatment at 85.7/8.57 μM (810P) and inter-assay under OV treatment at 85.7/8.57 μM (795-797-798P). * P = number of cell passages.

Table S2. Comparison between assays of EC_{50} and EC_{80} values of P-CTX3C and PbTx3 standards under two non-destructive O/V treatment (100/10 μM vs. 85.7/8.57 μM). WILCOXON TEST (*p*-values) (95% confidence level).

**EC_{50} P-CTX3C (fg/μL)**		**INTER-ASSAY** (100/10 μM)	**INTER-ASSAY** (85.7/8.57 μM)	**INTRA-ASSAY** (85.7/8.57 μM)
		(662-663-666P *)	(795-797-798P)	(810P)
**INTER-ASSAY** (100/10 μM)	(662-663-666P)		1.00	0.40
**INTER-ASSAY** (85.7/8.57 μM)	(795-797-798P)			0.40
**INTRA-ASSAY** (85.7/8.57 μM)	(810P)			

(**A**)

**EC_{80} P-CTX3C (fg/μL)**		**INTER-ASSAY** (100/10 μM)	**INTER-ASSAY** (85.7/8.57 μM)	**INTRA-ASSAY** (85.7/8.57 μM)
		(662-663-666P)	(795-797-798P)	(810P)
**INTER-ASSAY** (100/10 μM)	(662-663-666P)		1.00	0.40
**INTER-ASSAY** (85.7/8.57 μM)	(795-797-798P)			0.70
**INTRA-ASSAY** (85.7/8.57 μM)	(810P)			

(**B**)

**EC_{50} PbTX3 (fg/μL)**		**INTER-ASSAY** (100/10 μM)	**INTER-ASSAY** (85.7/8.57 μM)	**INTRA-ASSAY** (85.7/8.57 μM)
		(662-663-666P)	(795-797-798P)	(810P)
**INTER-ASSAY** (100/10 μM)	(662-663-666P)		1.00	0.40
**INTER-ASSAY** (85.7/8.57 μM)	(795-797-798P)			1.00
**INTRA-ASSAY** (85.7/8.57 μM)	(810P)			

(**C**)

**EC_{80} PbTX3 (fg/μL)**		**INTER-ASSAY** (100/10 μM)	**INTER-ASSAY** (85.7/8.57 μM)	**INTRA-ASSAY** (85.7/8.57 μM)
		(662-663-666P)	(795-797-798P)	(810P)
**INTER-ASSAY** (100/10 μM)	(662-663-666P)		1.00	0.40
**INTER-ASSAY** (85.7/8.57 μM)	(795-797-798P)			0.70
**INTRA-ASSAY** (85.7/8.57 μM)	(810P)			

(**D**)

A, Comparison of EC_{50} values of P-CTX3C standard between intra- and inter-assay. **B**, Comparison of EC_{80} values of P-CTX3C standard between intra- and inter-assay. **C**, Comparison of EC_{50} values of PbTx3 standard between intra- and inter-assay μM. **D**, Comparison of EC_{80} values of PbTx3 standard between intra- and inter-assay. * P = number of cell passages.

Table S3. Comparison between assays of LOD and LOQ values (ng P-CTX3C equivalents/g fish flesh) under non-destructive O/V treatment at 85.7/8.57 µM). WILCOXON TEST (*p*-values) (95% confidence level).

**INTER-ASSAY** (795-797-798P *)		**Fish sample**			
		**Cmic02**	**Cmic19**	**Emer05**	**Emer13**
**Fish sample**	**Cmic02**		0.7	0.2	0.4
	**Cmic19**			0.2	0.2
	**Emer05**				0.7
	**Emer13**				

(**A**)

**INTRA-ASSAY** (810P)		**Fish sample**			
		**Cmic02**	**Cmic19**	**Emer05**	**Emer13**
**Fish sample**	**Cmic02**		0.1	0.1	0.1
	**Cmic19**			0.1	0.1
	**Emer05**				0.1
	**Emer13**				

(**B**)

		**INTRA-ASSAY** (810P)			
	**Fish sample**	**Cmic02**	**Cmic19**	**Emer05**	**Emer13**
**INTER-ASSAY** (795-797-798P)	**Cmic02**	0.7			
	**Cmic19**		0.7		
	**Emer05**			0.7	
	**Emer13**				0.7

(**C**)

**INTER-ASSAY** (795-797-798P)		**Fish sample**			
		**Cmic02**	**Cmic19**	**Emer05**	**Emer13**
**Fish sample**	**Cmic02**		0.7	0.7	0.2
	**Cmic19**			0.2	0.2
	**Emer05**				0.2
	**Emer13**				

(**D**)

**INTRA-ASSAY** (810P)		**Fish sample**			
		**Cmic02**	**Cmic19**	**Emer05**	**Emer13**
**Fish sample**	**Cmic02**		0.1	0.2	0.1
	**Cmic19**			0.1	0.1
	**Emer05**				0.4
	**Emer13**				

(**E**)

		**INTRA-ASSAY** (810P)			
	**Fish sample**	**Cmic02**	**Cmic19**	**Emer05**	**Emer13**
**INTER-ASSAY** (795-797-798P)	**Cmic02**	0.4			
	**Cmic19**		0.4		
	**Emer05**			0.4	
	**Emer13**				0.4

(**F**)

A, Comparison of LOD values of the four fish samples obtained from intra-assay under non destructive O/V treatment 85.7/8.57 µM (810 P). **B**, Comparison of LOD values of the four fish samples obtained from inter-assays under non destructive O/V treatment 85.7/8.57 µM (795-797-798 P). **C**, Comparison of LOD values of the four fish samples obtained under non destructive O/V treatment 85.7/8.57 µM between intra-assay (810 P) and inter-assays (795-797-798 P). **D**, Comparison of LOQ values of the four fish samples obtained from intra-assay under non destructive O/V treatment 85.7/8.57 µM (810 P). **E**, Comparison of LOQ values of the four fish samples obtained from inter-assays under non destructive O/V treatment 85.7/8.57 µM (795-797-798 P). **F**, Comparison of LOQ values of the four fish samples obtained under non destructive O/V treatment 85.7/8.57 µM between intra-assay (810 P) and inter-assays (795-797-798 P). * P = number of cell passages.

3. Practical guide

3.1. Biological Materials and Reagents Required for the Implementation of the CBA-N2a

Table S4. List of biological materials.

Biological Materials
Mouse neuroblastoma (N2a) cell line (CCL-131) from the American Type Culture Collection (ATCC)
P-CTX3C (Institut Louis Malardé) (20 ng/mL)
PbTx-3 (Latoxan L89 02) (100 µg)
dc-STX (NRCC, ref. CRM-dc-STX-b) (65 µM in aqueous hydrochloric acid 3 mM)
STX (NRCC, ref. CRM-STX-f) (66.3 µM in aqueous hydrochloric acid 3 mM)
Chemical dry extracts from fish flesh samples (fraction LF90/10)

Table S5. List of reagents.

Reagents
RPMI medium 1640 with HEPES and without L-Glutamine (Gibco 42401)
Sodium pyruvate 100 mM (100X) (Gibco 11360-039)
GlutaMAX™-I (100X) (Gibco 35050-038)
Pen Strep (Penicillin at 5,000 Units/mL and Streptomycin at 5,000 µg/mL) (Gibco 15070-063)
Amphotericin B (250 µg/mL) (Gibco 15290-026)
Fetal Bovin Serum (Gibco 10270-106)
Thiazolyl Blue Tetrazolium Bromide (SIGMA M2128-250MG)
Dulbecco's Phosphate Buffered Saline without $CaCl_2$ and $MgCl_2$ (DPBS-1X)(Gibco 14190-094)
0.05% Trypsin-EDTA (1X) (Gibco 25300-062)
Dimethyl sulfoxide (DMSO) (SIGMA 34869-1L)
MeOH (LiChrosolv) (MERCK 1.06007.2500)
Ouabain octahydrate (SIGMA O3125-250MG)
Veratridine (SIGMA V5754-25MG)
Sterile distilled water
Sterile distilled water at pH 2 (HCl)
Phosphate Buffered Saline (PBS) (BioMérieux 75511)

3.2. Equipments and Technical Materials Required for the Implementation of the CBA-N2a

Table S6. List of equipments and materials.

Equipments	Materials
Laminar flow hood (MSC II)	Sterile 25 cm² or 75 cm² tissue culture flasks
Incubator (5% CO_2 and humidified atmosphere)	Sterile tissue Culture 96-well flat bottom microplates
Water bath (37 °C)	Sterilizable tanks
Refrigerator and freezer (–20 °C)	Sterile U bottom 96-well microtiter plates
Inverted microscope (phase-contrast optional)	Disposable cell counting slides
Sterilizer	Sterile pipettes (25, 10, 5, 2 and 1 mL)
50–1200 µL automated multichannel dispensing pipette (12 channels)	Disposable sterile tips adapted for automated and manual pipettes
10–100 µL automated multichannel dispensing pipette (8 channels)	microtiter tubes (1.1 mL)

20–300 µL manual multichannel pipette (12 channels)	Polypropylene tubes (50 and 15 mL)
Manual pipettes (10 to 1000 µL)	Glass or plastic vials
Automated pipettors	
Micro centrifuge	
Vortex	
Microplate reader with 570 nm filter	

3.3. Preparation of Stock Solutions

The stock solutions were prepared using the biological materials, reagents, equipements and materials listed in Tables S4 to S6 and established as following :

1. 10% FBS culture medium: complete RPMI medium 1640 with addition of 10% fetal bovine serum (FBS), 1% GlutaMAX, 1% sodium pyruvate, 1% Penicillin Streptomycin and 1% Amphotericin B.
2. 5% FBS culture medium: complete RPMI medium 1640 with addition of 5% FBS, 1% GlutaMAX, 1% sodium pyruvate, 1% Penicillin Streptomycin and 1% Amphotericin B.
3. 2% FBS culture medium: complete RPMI medium 1640 with addition of 2% FBS, 1% GlutaMAX, 1% sodium pyruvate, 1% Penicillin Streptomycin and 1% Amphotericin B.
4. Ouabain (20 mM) (O): 250 mg of Ouabain octahydrate were resuspended in 17.15 mL of sterile distilled water. Store at room temperature, avoiding direct exposure to light.
5. Veratridine (5 mM) (V): 25 mg of Veratrine were resuspended in 7.42 mL of pH 2 acidified sterile distilled water. Distribute in small 200 µL aliquots (to avoid repeated thawings of the stock solution) and store at −20 °C.
6. MTT (5 mg/mL): 250 mg of Thiazolyl Blue Tetrazolium Bromide were suspended in 50 mL of PBS. Distribute in 10 mL aliquots and store at −20 °C.
7. P-CTX3C toxin standard (20 ng/mL): 100 ng of P-CTX3C was diluted in 5 mL of DMSO. Store at −20 °C.
8. PbTx-3 toxin standard (100 µg/mL): 100 µg of PbTx-3 were resuspended in 1 mL of DMSO. Store at −20 °C.
9. STX toxin standard (21.4 µg/mL): STX was purchased in the form of a 0.5 mL ready-to-use solution at 66.3 ± 1.4 µmol/L in 3 mM aqueous hydrochloric acid. Store at −20 °C.
10. dc-STX toxin standard (24.7 µg/mL): dc-STX was purchased in the form of a 0.5 mL ready-to-use solution at 65 ± 1.8 µmol/L in 3 mM aqueous hydrochloric acid. Store at −20 °C.
11. Sample stock solution (10 mg/mL): determine the dry weight (DEW) of each LF 90/10 fraction extract (see Section 5.2. *Extraction procedure)* then resuspended each fraction in pure methanol in order to obtain a 10 mg/mL sample stock solution.

3.4. Definition of Six Key Viability Controls

The "DMSO control" is used to characterize the absorbance of the solvent (DMSO) used to dissolve N2a cell layers prior to measuring cell viability. The DMSO control absorbance (A_{DMSO}) is obtained by measuring the absorbance in the 2 peripheral wells of each microplate, in the absence of N2a cells. The mean absorbance value thus obtained is further deducted from all raw absorbance values, giving net absorbance data.

The "RCV or Reference Cell Viability control" is used to characterize the initial viability of N2a cells on day 2 after 26h of growth, when maximum cell density is reached in wells. The RCV control absorbance (A_{RCV}) is the mean of the absorbance values measured in n = 60 inner wells of a microplate.

The "COV^- control" is the counterpart of the RCV control and corresponds to the final viability of N2a cells at day 3, after 19h of additional culture in the absence of O/V treatment. The COV^- control absorbance (A_{COV^-}) is the mean of the absorbance values measured in n = 3 wells per microplate.

The "COV+ control" is used to check the final viability of N2a cells on day 3, after 19h of additional culture under O/V treatments. It also helps to verify the efficiency of the O/V treatment. The COV+ control absorbance (A_{COV^+}) is the mean of the absorbance values measured in *n* = 3 wells per microplate.

The "QCOV− control" is the quality control that serves to check for the final viability of N2a cells on day 3, after 19h of additional culture in the absence of O/V treatment and in the presence of VGSC acting toxins. The QCOV− control absorbance (A_{QCOV^-}) is the mean of the absorbance values measured in *n* = 3 wells per microplate.

The "QCOV+ control" is the quality control that serves to check for the final viability of N2a cells on day 3, after 19h of additional culture under O/V treatment and in the presence of VGSC acting toxins. The QCOV+ control absorbance (A_{QCOV^+}) is the mean of the absorbance values measured in *n* = 3 wells per microplate.

3.5. CBA-N2a Protocol

3.5.1. Cell seeding in microplates

The steps of this section require working under sterile conditions (e.g., laminar flow hood).

- Check cell layer has reached >90% confluence in culture flasks under an inverted microscope.
  NOTE: adapt size (25 vs. 75 cm²) and number of culture flasks to the number of microplates required for the CBA-N2a (minimum of 2 microplates).
- Pre-incubate the DPBS-1X, trypsin–EDTA solution, distilled sterile water and % FBS culture medium at 37 °C.
- Using a sterile tank and a 20–300 µL manual multipipette, distribute 200 µL of sterile water in the 36 peripheral wells of each 96-well microplate. Incubate at 37 °C.
- Using a pipette, remove culture medium from culture flasks without disturbing the cell layer. At the opposite side of the cell layer, add 5 mL of DPBS-1X, trypsin-EDTA solution per 75 cm² flask, and rinse the cell layer by slightly tilting the flask to cover the cell layer. Discard DPBS-1X.
  NOTE: this washing step removes traces of serum, calcium and magnesium that would inhibit the action of the dissociation reagent.
- Add 2 mL of trypsin-EDTA solution per 75 cm² flask, gently rock the flask to ensure complete coverage of the whole cell layer. Incubate for 1 min at 37 °C.
  NOTE: After each subculture step of the N2a cell line using trypsin-EDTA solution, the cell passage number is registered.
- Examine the detachment of the cell layer under the microscope. If less than 90% detachment is achieved, increase the incubation time checking for dissociation every 30 s.
- Detach the cells from the bottom of culture flasks by gentle taps on the flask, and add 5% FBS culture medium (1.5 mL/cm²) to stop the trypsin-EDTA effect.
- Homogenize the cell suspension up and down in the flask to ensure a single-cell suspension is obtained and pipet a small aliquot in plastique vial (<1 mL).
- Estimate cell density (*n* = 5 counts) from this aliquot using a counting slide. Adjust cell suspension to a seeding density of 2.5 to 3 × 10^5 cells/ mL in 5% FBS culture medium. Transfer the cell suspension into a sterile tank.
- Using a 20–300 µL manual multipipette, distribute 200 µL of this cell suspension into the 60 inner wells of each 96-well microplate.
  CAUTION: ensure the cell suspension is distributed evenly in wells by regular pipetting and ejecting the remaining cell suspension volume between deposits.
- Incubate microplate for 26 h at 37 °C, in a 5% CO_2 incubator.

3.5.2. Measuring N2a cell initial viability

The steps of this section do not require working under sterile conditions.

- Check cell layer has reached 100% confluence in all microplates under an inverted microscope (Figure S1a) and select one microplate.
- Proceed with a 1:6 dilution of the MTT stock solution in 2% FBS culture medium, to obtain a MTT solution at a final concentration of 0.83 mg/mL. Store at 37 °C until use, avoiding direct exposure to light.
  CAUTION: prepare enough solution for the MTT assay scheduled on day 3 (a total volume of 3.6 mL is required per microplate).
- Remove culture medium by overturning microplate, and dry wells by tapping on a dried absorbent paper to remove any residual medium.
- Using a 50–1200 µL automated multichannel dispensing pipette, distribute 60 µL of the 0.83 mg/mL MTT solution in sequence in each of the 60 inner wells of the microplate.
  CAUTION: position tips on the upper edge of the wells, using the smallest ejection speed to avoid cell detachment. Store the remaining MTT solution at +4 °C until day 3, avoiding direct exposure to light.
- Incubate the microplate for 45 min at 37 °C, in a 5% CO_2 incubator.
- Remove MTT solution by overturning microplate and dry wells by tapping on a dried absorbent paper.
  CAUTION: wear gloves and protection glasses.
- Check for the development of a dark blue color in wells.
- With a 50–1200 µL automated multichannel dispensing pipette, sequentially distribute 100 µL of DMSO in the 12 peripheral wells (B1-G1 and B12-G12) and the 60 inner wells seeded with N2a cells.
  NOTE: cellular lysis should take place immediately with the development of a dark purple color in the inner wells (Figure S1b). Use the faster ejection speed.
- Homogenize wells by gentle taps on the edges of the microplate.
  CAUTION: remove the microplate lid to avoid accidental splash.
- Read absorbance at 570 nm using a spectrophotometer.
- Save the raw absorbance data for further assay validation.

3.5.3. Ouabain/Veratridine (O/V) treatment

The steps of this section require working under sterile conditions (e.g., laminar flow hood).

- "O/V^- treatment" solution: prepare 6 mL of 2% FBS culture medium per microplate.
- "O/V^+ treatment" solution: prepare 6 mL of a mixed Ouabain and Veratridine (O/V) solution in 2% FBS culture medium per microplate, at the following concentrations:
- 60/6 to 100/10 µM final concentration in wells, which corresponds to non-destructive O/V treatment conditions when detecting VGSC activators (see Section 2.2.2 of the research paper).
- 270/27 to 300/30 µM final concentration in wells, which corresponds to destructive O/V treatment conditions when detecting VGSC inhibitors (see Section 2.2.2 of the research paper).
  NOTE: under O/V treatment, the final concentration of O/V in wells are 5% lower than the initial O/V concentration, taking into account the addition of 10 µL of toxin standard or sample extract in the 200 µL reaction volume.
- Remove the 5% FBS culture medium by overturning each microplate over a waste container and dry wells by tapping on dried adsorbent paper previously sterilized with 70% aqueous ethanol.
- Using an automated multichannel dispensing pipette, distribute 200 µL of the O/V^- treatment solution in n = 30 inner wells of the upper half of each microplate.
- Using an automated multichannel dispensing pipette, distribute 200 µL of the O/V^+ treatment solution in n = 30 inner wells of the bottom half of each microplate.

- Using an automated multichannel dispensing pipette, fill the 36 peripheral wells of each microplate with 200 µL of sterile water.
- Incubate microplate at 37 °C, in a 5% CO_2 incubator.
  CAUTION: avoid splashing medium onto the lid while handling the microplates.

3.5.4. Implementation of quality controls

- For COV^- and COV^+ controls, prepare 60 µL of 2% FBS culture medium per microplate.
- For $QCOV^-$ and $QCOV^+$ controls, prepare 60 µL of the appropriate toxin standard stock solution in 2% FBS culture medium, at a final concentration close to EC_{50}.
- Using a 10–100 µL automated multichannel dispensing pipette, add 10 µl of each control solution in *n* = 3 wells, under OV^- and OV^+ conditions, prior to qualitative screening or composite toxicity analyses (Figure S2).

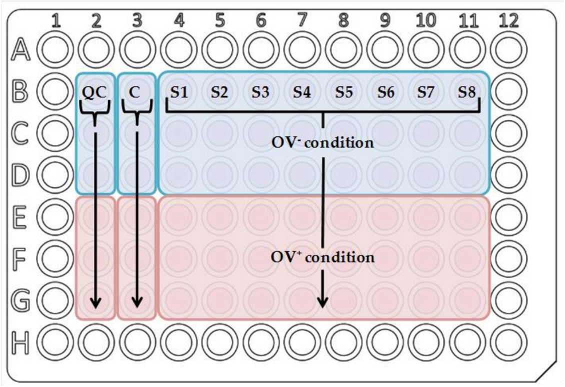

(**a**)

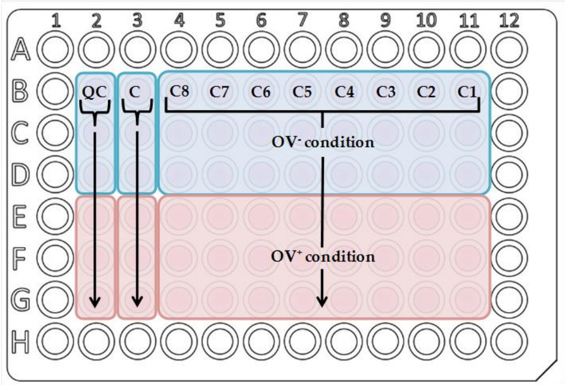

(**b**)

Figure S2. Suggested layouts on a 96-wells microplate at day 2 of the CBA-N2a. (**a**) qualitative screening of eight distinct samples (S1 to S8) per microplate at a unique concentration just below the MCE (previously established as explained in Section 2.3.2); (**b**) composite toxicity analyses performed on one sample (toxin standard or biological extract) per microplate, tested at eight distinct concentrations (C1 to C8). Quality check and viability controls are distributed in wells (B2-G2) and (B3-G3), respectively, in OV^- ($QCOV^-$ and COV^-) and OV^+ ($QCOV^+$ and COV^+) conditions, each condition tested in triplicate (*n* = 3 wells).

3.5.5. For the qualitative screening of biological samples (each sample tested at a unique concentration

- In a U bottom 96-well microtiter plate, add 2 µL of each sample stock solution in 98 µL of 2% FBS culture medium (1:50 dilution). Homogeneize the solution.
  CAUTION: sample stock solutions must be prepared in advance, stored at −20 °C and brought to room temperature prior to CBA-N2a analyses. Change tips between deposits and avoid foam formation during mixing.
- Using a 10–100 µL automated multichannel dispensing pipette, add 10 µL of each diluted sample solution to 200 µL of O/V^- and O/V^+ treatments to reach a final concentration of 9524 pg/µL in wells (Figure S2a).
  NOTE: using this screening format, eight different samples (S1 to S8) can be tested in parallel per microplate, at a concentration of 9524 pg/µL, i.e., just below the MCE (established at 10,000 pg/µL for LF90/10 in this study for CTX detection) (see Section 2.3.2 of the research paper).
  CAUTION: the MCE value is likely to vary according to the extraction protocol used and the type of fraction tested, and should therefore be determined in each laboratory prior to CBA-N2a analyses.
- Incubate microplates overnight for 19h, at 37 °C, in a 5% CO_2 incubator.
  CAUTION: Avoid splashing medium onto the lid while handling microplates.

3.5.6. For the composite toxicity estimation of biological samples (each sample tested at eight distinct concentrations)

- In a U bottom 96-well microtiter plate, prepare at least 200 µL of initial concentration (C'1) by diluting both the toxin standard and sample stock solutions in 2% FBS culture medium, using a 1:50 dilution for P-CTX3C, PbTx3 and sample stock solutions, a 1:12.5 dilution for STX stock solution and a 1:2.5 dilution for dc-STX stock solution.
  CAUTION: bring all stock solutions to room temperature before use.
- Using a 20-300 µL manual multipipette and a U bottom microplate, prepare an eight-points serial 1:2 dilution by first transferring 100 µL of initial concentration (C'1) into 100 µL of 2% FBS culture medium, then 100 µL of the resulting initial concentration (C'2) in 100 µL of 2% FBS culture medium and so on.
  CAUTION: change tips between dilutions and avoid foam formation during mixing.
- Using 10-100 µL automated multichannel dispensing pipette, add 10 µL of each initial concentration in triplicate wells to 200 µL of O/V^- and O/V^+ treatments for testing final concentrations (C1 to C8) in a final volume of 210 µL (Figure S2b). In this test format, only one sample can be tested per microplate, at eight distinct concentrations, each concentration run in triplicate.
  CAUTION: position tips at the top and in the middle of wells and use the lowest ejection speed.
- Incubate microplate overnight for 19h, at 37 °C, in a 5% CO_2 incubator.
  CAUTION: Avoid splashing medium onto the lid while handling microplates.
- Check the microplates after 19–22 h incubation. Culture medium color in controls wells should be: yellow in OV^- conditions and pink in OV^+ conditions.
- Warm up MTT solution prepared on day 2 in a liquid bath, at 37 °C, avoiding direct exposure to light.
- Remove culture medium by overturning microplate, and dry wells by tapping on an absorbent paper.
- Measure cell viability as in section 3.5.2 for each microplate.

3.6. CBA-N2a Data Analysis

3.6.1. Validation of the CBA-N2a results by means of appropriate viability controls

- N2a cell initial viability

For the microplate used for RVC control, determine the mean absorbance value measured in the 12 wells of DMSO control (A_{DMSO}). Determine the mean net absorbance for the RCV control (A_{RCV}) by deducting A_{DMSO} from the raw absorbance values measured in the 60 inner wells of the RCV control microplate. $A_{RCV} \geq 1$ indicates optimal initial cell viability (Table S7).

- N2a cell final viability

For each microplate used for qualitative screening or composite toxicity estimation, determine the mean absorbance value measured in the 12 wells of DMSO control (A_{DMSO}). Deduct A_{DMSO} from raw absorbance data of COV^-, COV^+, $QCOV^-$ and $QCOV^+$ viability controls (n = 3) to obtain corresponding mean net absorbance values (A_{COV^-}, A_{COV^+}, A_{QCOV^-}, A_{QCOV^+}). Expected values should be as in Table S7 to enable further validation of the assay data.

Table S7. Expected results for viability controls to enable validation of the assay.

**Controls (OV Conditions)**	**Expected net Absorbance Values**	**Conclusion**
RCV (before O/V treatment)	$A_{RCV} \geq 1$	high initial cell viability
COV^{-} (without O/V treatment)	$A_{COV^{-}} \geq 1$	preservation of the initial cell viability
COV^{+} (non-destructive O/V treatment)	$A_{COV^{+}} \geq 1$	preservation of the initial cell viability
COV^{+} (destructive O/V treatment)	$A_{COV^{+}} = 0.1 \pm 0.1$	elimination of initial viability
$QCOV^{-}$ (without O/V treatment)	$A_{QCOV^{-}} \geq 1$	no activity on VGSCs
$QCOV^{+}$ (non-destructive O/V treatment)	$A_{QCOV^{+}} \geq 0.2 \times A_{COV^{+}}$ (*) and $A_{QCOV^{+}} \leq 0.8 \times A_{COV^{+}}$ (*)	Presence of VGSCs activators
$QCOV^{+}$ (destructive O/V treatment)	$A_{QCOV^{+}} \geq (0.2 \times A_{RCV} + 0.8 \times A_{COV^{+}})$ (*) and $A_{QCCOV^{+}} \leq (0.8 \times A_{RCV} + 0.2 \times A_{COV^{+}})$(*)	Presence of VGSCs inhibitors

(*) see Section 2 below for definition.

3.6.2. Qualitative screening analysis

Determine the mean net absorbance value (A) for each dry extract (LF90/10) tested at a unique concentration (each point run in triplicate), in OV^{-} and OV^{+} conditions. Results are further interpreted with reference to the decision (DL) and confirmation (CL) limits of the assay. Figures S3 and S4 explain how the DL and CL are established, depending on the targeted toxin group (i.e., VGSC activators or inhibitors).

- In the absence of O/V treatment.

  No VGSC activation or inhibition should be detected.

  If $A \geq A_{COV^{-}}$, cell viability enhancement is probably due to the dry extract.

  If $A \leq A_{COV^{-}}$, the presence of other unknown toxins causing a decrease in cell viability should be suspected.

- Under non-destructive O/V treatment (for the detection of VGSC activators).

  Here, DL and CL correspond to the detection of 20% and 80% mortality induced by the sample as compared to the final cell viability observed in the absence of toxin, i.e., $DL = 0.8 \times A_{COV^{+}}$ and $CL = 0.2 \times A_{COV^{+}}$, respectively (Figure S3).

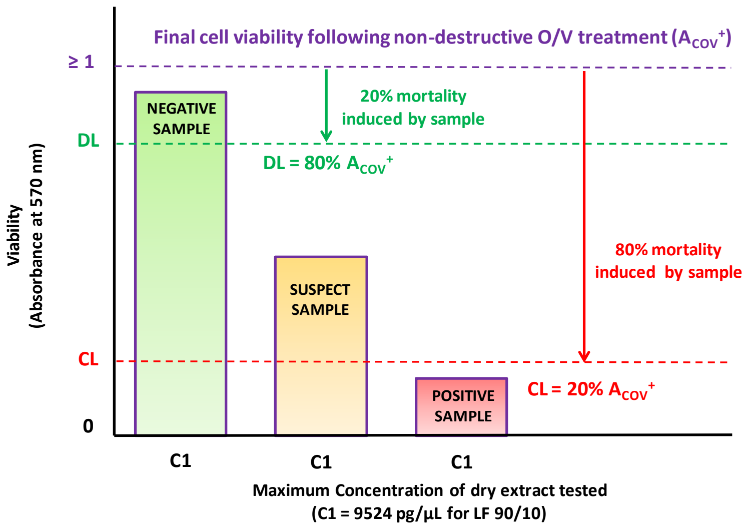

Figure S3. Detection (DL) and confirmation (CL) limits adopted in qualitative screening analysis of samples tested for the presence of VGSC activators (under non-destructive O/V treatment). Samples are tested at a unique concentration (C1) adjusted just below the MCE. The histograms illustrate the ranges of absorbance values indicative of a negative, suspect and positive sample, respectively.

If A ≤ CL, POSITIVE sample is suspected signing the presence of quantifiable amounts of VGSC activators.

If CL ≤ A ≤ DL, SUSPECT sample is suspected signing the presence of trace amounts of VGSC activators.

If A ≥ DL, NEGATIVE sample is suspected.

- Under destructive O/V treatment (for the detection of VGSC inhibitors)

Here, DL and CL correspond to a restoration of 20% and 80% of cell viability induced by the sample as compared to the initial viability of bioassay, i.e., DL = (0.2 × A_{RCV} + 0.8A_{COV}^+) and CL = 0.8 × A_{RCV} + 0.2 × A_{COV}^+), respectively (Figure S4). Note that with A_{COV}^+ being around 0.1, DL and CL values are close to 0.2 × A_{RCV} and 0.8 × A_{RCV}, respectively.

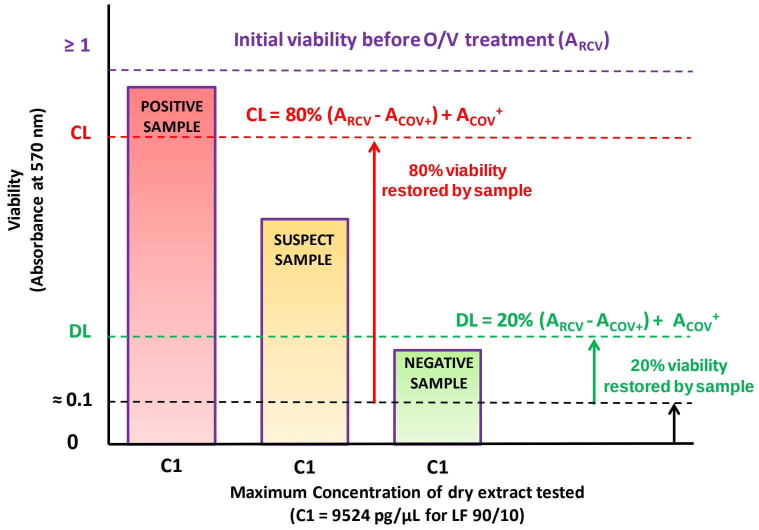

Figure S4. Detection (DL) and confirmation (CL) limits adopted in qualitative screening analysis of samples tested for the presence of VGSC inhibitors (under destructive O/V treatment). Samples are

adjusted just below the MCE. The histograms illustrate the ranges of absorbance values indicative of a negative, suspect and positive sample, respectively.

If A ≥ CL, POSITIVE sample is suspected signing the presence of quantifiable amounts of VGSC inhibitors.

If DL ≤ A ≤CL, SUSPECT sample is suspected signing the presence of trace amounts of VGSC inhibitors.

If A ≤ DL, NEGATIVE sample is suspected.

NOTE: samples found "suspect" and "positive" following this screening test can further be tested using an eight-points serial dilution 1:2, in order to establish a full dose-response curve for toxin quantification. For suspect samples, the first concentration to test could be the one corresponding to a 1:10 dilution instead of 1:50, for a quick confirmation of either matrix effect or proven toxicity.

3.6.3. Dose response curves

Determine the mean net absorbance value (A) measured for each dry extract (LF90/10 fraction) tested at eight distinct concentrations (C1 to C8, each point run in triplicate), in OV^- and OV^+ conditions. Plot net absorbance data (Y-axis) against toxin and dry extract concentrations (X-axis, in logarithmic scale) with 4PL using GraphPad Prism software version 8.1.2 (GraphPad, San Diego, CA, USA) to generate full dose-response curves. Detection of a specific activation or inhibition of VGSCs is interpreted as follows:

- In the absence of O/V treatment

  No sigmoid dose-response curve is obtained.
- Under non-destructive O/V treatment (for the detection of VGSC activators)

  In the presence of VGSC activators, a sigmoidal dose-response curve with a positive Hillslope is obtained with $0 \leq A_{C1-C8} \leq A_{COV+}$ (Figure S5).

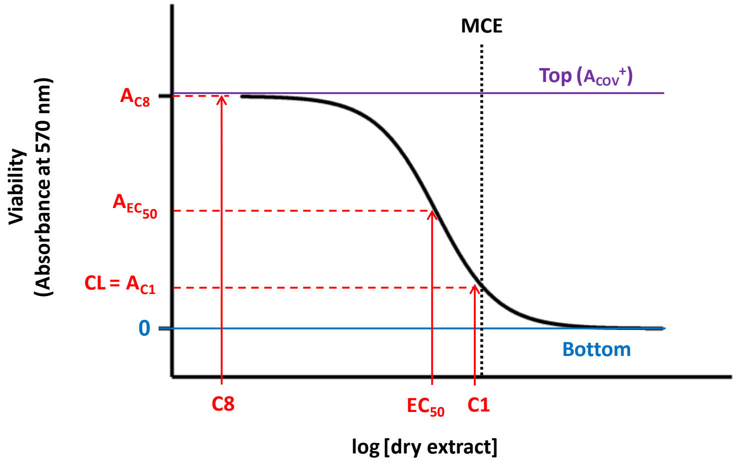

Figure S5. Typical dose-response curve expected with a sample positive for VGSC activators, tested at eight distinct final concentrations (C1–C8) adjusted to fall below the MCE. The effective concentration of dry extract inducing a viability half way (50%) between the basal (Bottom) and the maximal (Top) values of the curve (EC_{50}) is also indicated.

- Under destructive O/V treatment (for the detection of VGSC inhibitors)

  In the presence of VGSC inhibitors, a sigmoidal dose-response curve with a negative Hillslope is obtained, with $A_{COV+} \leq A_{C1-C8} \leq A_{RCV}$ (Figure S6).

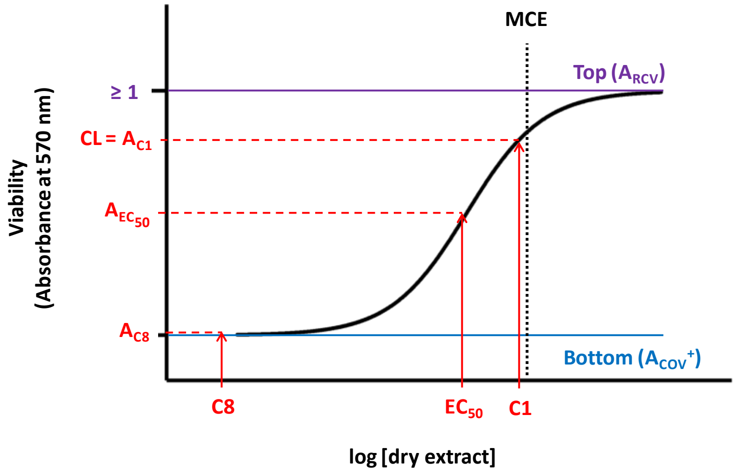

Figure S6. Typical dose-response curve expected with a sample positive for VGSC inhibitors, tested at eight distinct final concentrations (C1–C8) adjusted to fall below the MCE. The effective concentration of dry extract inducing a viability half way (50%) between the basal (Bottom) and the maximal (Top) values of the curve (EC_{50}) is also indicated.

3.6.4. Composite toxicity estimations

For each dose-response curve established for both the selected toxin standard and toxic samples, determine the effective concentration inducing a response half way (50%) between the bottom and top absorbance values, or EC_{50}. Ensure a full dose-response curve is obtained by properly adjusting C1 to allow proper determination of this EC_{50} value, for more accurate and reliable results.

The composite toxicity in fish dry extracts (T), expressed in ng P-CTX3C eq/mg, is determined by comparing the EC_{50} values of P-CTX3C and fish dry extracts determined in the same experiment, using the following equation:

$$\text{T (ng of toxin standard eq/mg)} = EC_{50} \text{ of toxin standard (fg/}\mu\text{L)}/EC_{50} \text{ of dry extract (pg/}\mu\text{L)} \quad (1)$$

The composite toxicity in biological samples (Q), expressed in ng P-CTX3C eq/g of fish flesh, is determined using the following equation:

$$\text{Q (ng of toxin standard eq/g)} = \text{T} \times \text{(DEW)/FW)} \quad (2)$$
